# Evolution of egalitarian social norm by resource management

**DOI:** 10.1371/journal.pone.0227902

**Published:** 2020-01-30

**Authors:** Xiaofeng Wang, Xiaojie Chen, Long Wang

**Affiliations:** 1 Department of Automation, School of Information Science & Technology, Donghua University, Shanghai, China; 2 Engineering Research Center of Digitized Textile & Apparel Technology, Ministry of Education, Donghua University, Shanghai, China; 3 School of Mathematical Sciences, University of Electronic Science and Technology of China, Chengdu, China; 4 Center for Systems and Control, Peking University, Beijing, China; Wenzhou University, CHINA

## Abstract

Social organizations, especially human society, rely on egalitarian social norm, which can be characterized by high levels of fairness, empathy and collective conformity. Nevertheless, the evolution of egalitarian social norm remains a conundrum, as it suffers the persistent challenge from individual self-interest. To address this issue, we construct an evolutionary game theoretical model by employing the Ultimatum Game, in which rational individuals are able to perform resource management. We show that resource management drives a population evolving into an oscillatory state with high equilibrium degrees of fairness, empathy and collective conformity and thus constitutes a key mechanism for the evolution of egalitarian social norm in social dilemma situations. Specifically, it results in (1) the formation of egalitarian social norm from diverse individual norms, (2) the emergence of egalitarian social norm in a selfish and unfair world, and (3) the maintenance of egalitarian social norm despite the presence of norm violators. The constructive role of resource management is explained by a mean-field analysis revealing that resource management can effectively enlarge the attraction basin of egalitarian norms or even change the dynamical property of the mini Ultimatum Game from bistability between egalitarian norms and less egalitarian norms to complete-dominance of egalitarian norms over less egalitarian norms. Furthermore, we find that the capacity of resource management can be evolutionarily selected by a coevolution between egalitarian social norm and resource management. Our study suggests that efficiency and equity are linked to each other.

## Introduction

Social norms are ubiquitous in the real world and can be observed at different periods of social organizations, ranging from pre-industrial to post-industrial societies [1, 2]. Most importantly, the maintenance of social order, cohesion and stability requires the constraint of social norms [3, 4]. Particularly, as a subset of social norms, egalitarian social norm plays a vital role in strengthening as well as explaining the prevalence of large-scale cooperation in human society [5, 6]. Our definition of egalitarian social norm comprises triple contents: high levels of fairness, empathy and collective conformity. Fairness means that people prefer equality and are willing to pay a price to achieve more equitable outcomes, i.e., inequity aversion [7]. Empathy means that people make offers which themselves are prepared to accept [8–10]. Collective conformity means that people conform to wildly shared norms of how one ought to behave in a given situation [1, 3, 11]. Although its significant meaning and function for human society, egalitarian social norm is also vulnerable and permanently challenged by the selfishness of social members. That is, enforcement of egalitarian social norm is often costly: norm enforcers need to bear a cost to punish norm violators. Self-interested individuals bent on maximizing income therefore should not punish, which accordingly causes the collapse of egalitarian social norm. Then how can we understand the evolution of egalitarian social norm in a population of unrelated individuals [2, 12]?

The conflicted scenarios of egalitarian social norm between constructive (i.e., social order, cohesion and stability) and destructive powers (i.e., individual selfishness and greediness) can be conveniently modeled as social dilemma situations. Especially, the Ultimatum Game [13] can succinctly capture the fundamental essence of social dilemmas related to egalitarian social norm. In this simple game, two players are offered a chance to split a batch of resources (or a sum of money). One player proposes a division of the resources between them, and the other one can either accept or reject it. In the case of rejection, neither player receives anything, whereas in the case of acceptance, the proposed division is implemented. Based on canonical assumption of selfish rationality, both game theory and conventional evolutionary game theory [14, 15] make the prediction that individuals follow the unfair norm: responders should accept any nonzero offer, and proposers should offer the smallest possible share. To evaluate these game-theoretical predictions, lots of behavioral experiments have been conducted on the Ultimatum Game [7, 13, 16–20] (for a review, see [21] or [22] more recently). Although considerable quantitative variations across these empirical studies, the robust qualitative observations deviating from the rational self-interest are: (1) many responders choose to reject low offers (about half of them reject offers below 30% of the total sum), and (2) many proposers offer more than minimum amount required to avoid rejection (the majority of proposers offer 40 to 50% of the total sum). Until recently, a number of explanations have been provided to overcome the discrepancy between theoretical predictions and experimental observations. Some behavioral experiments demonstrated that most humans are not purely self-centered but have other-regarding preferences, which can be considered in the definitions of utility functions [7, 21, 23, 24]. Other works have shown that the preference of people towards fairness may be due to the failure of “seizing the moment” (i.e., after the game there will be no further interactions between two players) [7, 18, 25, 26]. In the framework of evolutionary game theory, theoretical studies indicate that asymmetric mutation structure [27], small group size [28], reputation [29], empathy [9], randomness [30, 31] and population structure [32–37] play a vital role in the evolution of fairness in the Ultimatum Game (see [38] for a review).

On the other hand, individuals, especially human kind, would like to perform resource management when they face with resource allocation problems in reality. By performing resource management, individuals can effectively deploy and assign resources so as to achieve the goal of efficient utilization. However, until now, little is known about the significance of resource management for the evolution of egalitarian social norm. In what follows, we integrate a model of resource management and model the evolution of egalitarian social norm in the Ultimatum Game following a game-theoretical way [39, 40] (see **Model Definition** in Methods section). This is motivated by the fact that individuals prefer more material benefits, e.g., a larger amount of resources or a larger sum of money. In the Ultimatum Game, failure to reach an agreement is a waste of resources, which is detrimental for both the proposer and the responder, while the achievement of an agreement can be regarded as an efficient utilization of resources, which is beneficial for both of them. To improve their incomes, individuals are often willing to perform resource management. In our model of resource management, both parties (proposer and responder) incline to allocate more resource to the deal, where the split succeeded in the previous round, but tend to assign less resource to the deal, where the split failed in the previous round. In fact, there are numerous examples falling into this category of resource management scheme. Let us take trade relationships between China and US as an example. China-US trade disputes in certain trading sectors, such as China-US iron and steel trade frictions and China-US textile and clothing trade frictions, hurt the interests of both nations. For instance, due to the drastic trade tensions between China and US, the average trade (≈1.01 billion dollars) in iron and steel sector between 2009 and 2011 decreased by 57% in comparison with that (≈2.35 billion dollars) between 2006 and 2008 [41]. However, the total China-US trade still increased by 12% in the meantime (from 316 to 354 billion dollars) [41], which indicates that the trade amounts in other non-controversial trade sectors were still increasing. This enlarges the pie of China-US shared interests and thus benefits the economies of both countries.

Interestingly, we show that natural selection favors the evolution of egalitarian social norm whenever resource management is introduced. To be specific, it can lead to (1) the formation of egalitarian social norm from diverse individual norms, (2) the emergence of egalitarian social norm in a selfish and unfair world, and (3) the maintenance of egalitarian social norm despite the presence of norm violators. Furthermore, we find that the capacity of resource management can coevolve with egalitarian social norm.

## Results

In this article, we focus on how resource management (i.e., the intensity of resource management Δ) affects the evolution of egalitarian social norm, the definition of which includes fairness (i.e., the mean offer level $\bar{p}$ and the mean acceptance level $\bar{q}$), empathy (i.e., the mean empathy level $\bar{e}$) and collective conformity (i.e., the conformity level $\bar{c}$; see **Population Variables** in Methods section).

### Evolution of egalitarian social norm by resource management

**Fig 1:**
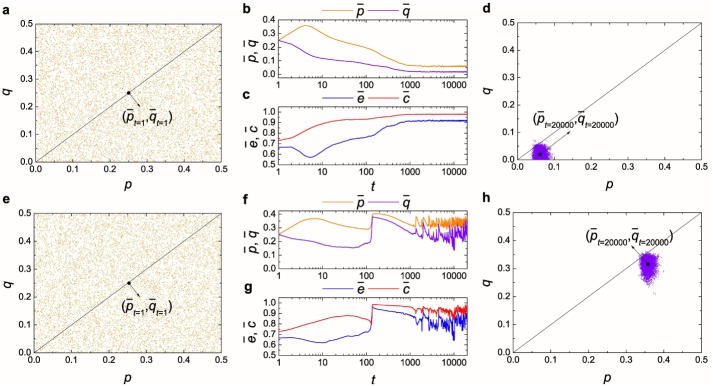
Resource management determines the formation of egalitarian social norm from diverse individual norms. The top row is for the intensity of resource management Δ = 0, and the bottom row is for the intensity of resource management Δ = 1. (**a**, **e**) Characteristic norm distribution of the initial state (**a**: ${\bar{p}}_{t=1}\approx 0.25$, ${\bar{q}}_{t=1}\approx 0.25$, ${\bar{e}}_{t=1}\approx 0.665$, ${\bar{c}}_{t=1}\approx 0.729$; **e**: ${\bar{p}}_{t=1}\approx 0.253$, ${\bar{q}}_{t=1}\approx 0.25$, ${\bar{e}}_{t=1}\approx 0.666$, ${\bar{c}}_{t=1}\approx 0.728$). (**b**, **c**, **f**, **g**) Time evolution of four population variables. (**d**, **h**) Characteristic norm distribution of the equilibrium state (**d**: ${\bar{p}}_{t=20,000}\approx 0.061$, ${\bar{q}}_{t=20,000}\approx 0.02$, ${\bar{e}}_{t=20,000}\approx 0.917$, ${\bar{c}}_{t=20,000}\approx 0.979$; **h**: ${\bar{p}}_{t=20,000}\approx 0.358$, ${\bar{q}}_{t=20,000}\approx 0.315$, ${\bar{e}}_{t=20,000}\approx 0.915$, ${\bar{c}}_{t=20,000}\approx 0.973$). Note the logarithmic scale on *x*-axis in (**b**, **c**, **f**, **g**). Parameter settings: exploration rate *μ* = 0, noise level *K* = 0.1 and learning error range *ε* = 5 × 10^−3^.

We begin by investigating the formation of egalitarian social norm from diverse individual norms via resource management. The typical collective behavior of a population on a fully connected network is presented in Fig 1, when resource management is absent (i.e., Δ = 0; see the top row of Fig 1) and present (i.e., Δ = 1; see the bottom row of Fig 1). The left and right columns of Fig 1 respectively display the characteristic norm distribution of the initial and the stationary state, and the middle column of Fig 1 shows the time evolution of fairness, empathy and collective conformity. The evolution displayed in Fig 1 starts from a population of players with diverse individual norms (that is, the individual norms are randomly distributed in the *p* − *q* parameter space; ${\bar{p}}_{t=1}\approx 0.25$, ${\bar{q}}_{t=1}\approx 0.25$, ${\bar{e}}_{t=1}\approx 0.665$, ${\bar{c}}_{t=1}\approx 0.729$ for Δ = 0; ${\bar{p}}_{t=1}\approx 0.253$, ${\bar{q}}_{t=1}\approx 0.25$, ${\bar{e}}_{t=1}\approx 0.666$, ${\bar{c}}_{t=1}\approx 0.728$ for Δ = 1; see Fig 1**a** and 1**e**). At the early stage of evolution, the mean offer of the population increases whereas the mean acceptance threshold decreases, as it is the best response to random individual norms [32]. Meanwhile, we can also observe the decline of the average empathy level of the population and the rise of the average conformity level (see Fig 1**b**, 1**c**, 1**f** and 1**g**). Later on, natural selection guides the contrary evolutionary processes for Δ = 0 and Δ = 1 (compare Fig 1**b**, 1**c** and 1**d** with Fig 1**f**, 1**g** and 1**h**). The absence of resource management creates an empathetic and coherent state of the population, but one that is locked in selfish behavior (${\bar{p}}_{t=20,000}\approx 0.061$, ${\bar{q}}_{t=20,000}\approx 0.02$, ${\bar{e}}_{t=20,000}\approx 0.917$, ${\bar{c}}_{t=20,000}\approx 0.979$; see Fig 1**d**). Unfairness is, in this case, the only behavior that tends to spread over the whole population, as fair individuals imitate more successful rational ones. However, under the presence of resource management, fairness and empathy prevail after some relaxation time, reaching a stationary state where the population is fair as well as empathetic, and individuals tend to conform to the egalitarian norms (${\bar{p}}_{t=20,000}\approx 0.358$, ${\bar{q}}_{t=20,000}\approx 0.315$, ${\bar{e}}_{t=20,000}\approx 0.915$, ${\bar{c}}_{t=20,000}\approx 0.973$; see Fig 1**h**). That is, egalitarian social norm forms among individuals with diverse individual norms.

Furthermore, one can see that there are perpetual oscillations around the equilibrium after the whole system enters into the stationary state (see Fig 1**f**). Such an interesting phenomenon can be understood as follows. Suppose that the population has already reached the egalitarian state with the prevalence of individual norm $\left[\bar{p},\bar{q}\right]$ (see Fig 1**h**). Then such an egalitarian norm will be invaded by individual norms [*p*, *q*] with their offer levels *p* and acceptance thresholds *q* satisfying $\bar{p}\geq p\geq \bar{q}$ and $q\leq \bar{p}$. Thus we can see the temporary decrease of offer level $\bar{p}$, which induces the transient decline of acceptance level $\bar{q}$ in the equilibrium (see Fig 1**f**). The decrease of offer levels of some individuals would cause numerous conflicted events in the population. That is, individuals with egalitarian norms will reject the offers proposed by individuals with less egalitarian norms. In the presence of resource management, individuals with egalitarian norms can defeat ones with less egalitarian norms (for details see the analysis of a mini Ultimatum Game below). Therefore, the average offer level $\bar{p}$ and average acceptance level $\bar{q}$ of the population would rebound (see Fig 1**f**). In addition, we can also find that the population would never reach a fully egalitarian state (i.e., $\bar{p}=0.5$ and $\bar{q}=0.5$) (see Fig 1**f**). When the offer level of an individual norm approaches closer to 0.5, the evolutionary advantage of such a norm over other less egalitarian norms would gradually lost. In fact, if the offer level of an individual norm is 0.5, this norm would never win over other self-compatible norms (a draw at best). This is true no matter the mechanism of resource management is present or not (for details see the analysis of a mini Ultimatum Game below). Hence, no individual will adopt this kind of disadvantageous norms, and the whole population would never reach and stay in the fully egalitarian state.

**Fig 2:**
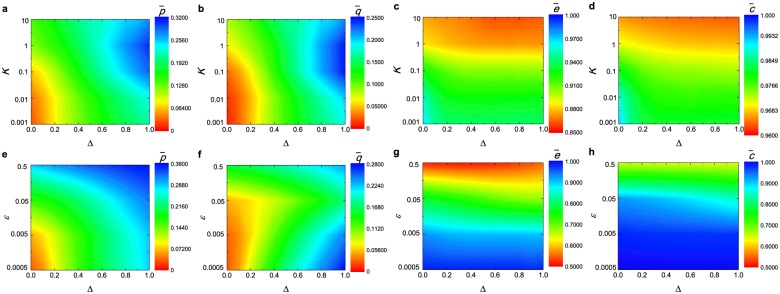
Equilibrium state of a population from diverse individual norms. The top (bottom) panels display the asymptotic values of four population variables describing the equilibrium state of the population as a function of Δ and the noise *K* (the learning error range *ε*): (**a**, **b**, **e**, **f**) fairness (i.e., $\bar{p}$ and $\bar{q}$), (**c**, **g**) empathy (i.e., $\bar{e}$) and (**d**, **h**) collective conformity (i.e., $\bar{c}$). All other model setups are the same as those used in Fig 1.

We systematically explore the formation of egalitarian social norm on fully connected networks by studying the parameter dependence of the stationary collective behavior of the model. The parameter space presented in the top row of Fig 2 is spanned by Δ and the noise level *K* whereas that presented in the bottom row of Fig 2 is spanned by Δ and the learning error range *ε*. Our results reveal that, whenever resource management is considered (i.e., Δ > 0), the formation of egalitarian social norm can be facilitated, regardless of the noise level (see Fig 2**a**, 2**b**, 2**c** and 2**d**) and learning error range (see Fig 2**e**, 2**f**, 2**g** and 2**h**). By comparison, a population of individuals without capability of resource management (i.e., Δ = 0) is unable to achieve such degree of social equity.

In S1 Text, we further demonstrate that resource management can even lead to the emergence of egalitarian social norm in a selfish and unfair world and the maintenance of egalitarian social norm despite the presence of norm violators. In addition, we also confirm that the presented results are robust with respect to (1) the detailed network topology, (2) the updating pattern, (3) the evolutionary dynamics, (4) the definition of the Ultimatum Game, and (5) the norm distribution range (see full details in S1 Text).

To reveal the beneficial role of resource management in the evolution of egalitarian social norm, we study a mini Ultimatum Game by using replicator dynamics [42]. This simplified model considers discrete norms, yet incorporates key features of the full game with its continuum of norms: (1) Individuals tend to adopt the norm they perceive to be that of the local optimum. (2) The conflicts between individuals with different norms are retained during their interactions with others. In our mini Ultimatum Game, only two individual norms are available in the population, i.e., an egalitarian norm (i.e., a self-compatible and generous norm) *IN*_2_ = [*p*_2_, *q*_2_] with $0\leq p_{2},q_{2}\leq \frac{1}{2}$ and a less egalitarian one (i.e., a self-compatible but less generous norm) *IN*_1_ = [*p*_1_, *q*_1_] with $0\leq p_{1},q_{1}\leq \frac{1}{2}$. We say that the norm *IN*_2_ is more generous than *IN*_1_ if *p*_2_ > *p*_1_ and *p*_2_ ≥ *q*_1_. Meanwhile, a norm *IN* = [*p*, *q*] is said to be self-compatible if *p* ≥ *q*. Self-incompatible norms are omitted, because they get eliminated in the mean-field limit anyway [29]. Hence the following three cases of the mini Ultimatum Game should be considered:

1. $0\leq q_{1}\leq q_{2}\leq p_{1}<p_{2}\leq \frac{1}{2}$.
2. $0\leq q_{2}<q_{1}\leq p_{1}<p_{2}\leq \frac{1}{2}$.
3. $0\leq q_{1}\leq p_{1}<q_{2}\leq p_{2}\leq \frac{1}{2}$.

In cases 1 and 2, both the payoff of a player with norm *IN*_1_ and that of a player with norm *IN*_2_ are invariant with Δ, irrespective of the composition of the population. Therefore, we are mainly concerned with case 3. In this case, the interaction between a player with norm *IN*_2_ and a player with norm *IN*_1_ can be described by the following 2 × 2 payoff matrix,

$$\begin{array}{c} \begin{array}{cc} & \begin{array}{cc} IN_{2} & IN_{1} \end{array}\\ \begin{array}{c} IN_{2}\\ IN_{1} \end{array} & \begin{array}{cc} a & b\\ c & d \end{array} \end{array}=\begin{array}{cc} & \begin{array}{cc} IN_{2} & IN_{1} \end{array}\\ \begin{array}{c} IN_{2}\\ IN_{1} \end{array} & \begin{array}{cc} 1 & 1-p_{2}\\ p_{2} & 1 \end{array} \end{array}. \end{array}$$ (1)

**Fig 3:**
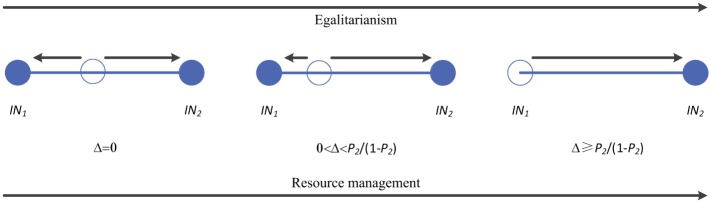
Egalitarian norm dominates in the mini Ultimatum Game if individuals can perform resource management. There are two norms: the egalitarian norm (i.e., the self-compatible and generous norm) *IN*_2_ = [*p*_2_, *q*_2_] offers and accepts high shares, and the less egalitarian one (i.e., the self-compatible but less generous norm) *IN*_1_ = [*p*_1_, *q*_1_] offers and accepts low shares. The figure shows the flow of evolutionary dynamics on the simplex *S*_2_ [42]. The solid dot represents the stable fixed point, while the hollow one the unstable fixed point.

Here a player with norm *IN*_2_ obtains *a* = 1 from another player with norm *IN*_2_, but *b* = 1 − *p*_2_ from a player with norm *IN*_1_. Similarly, a player with norm *IN*_1_ obtains *c* = *p*_2_ from a player with norm *IN*_2_, but *d* = 1 from another player with the same norm. As *a* > *c* and *b* < *d*, both norms are best replies to themselves, and thus there is an unstable fixed point between the norm *IN*_1_ and *IN*_2_ in the evolutionary game (i.e., bistability between the norm *IN*_1_ and *IN*_2_). Furthermore, since *a* = *d* and *b* ≥ *c*, the norm *IN*_2_ is risk-dominant over *IN*_1_ and has a larger basin of attraction than *IN*_1_ does. In S1 Text, we show that resource management is able to help the egalitarian norm *IN*_2_ to enlarge the basin of attraction or even able to dominate the less egalitarian norm *IN*_1_ (see Fig 3). In fact, the average payoff of players with norm *IN*_2_ and that of players with *IN*_1_ suggest that resource management introduces a simple transformation of the payoff matrix (see Eqs. S3 and S4 in S1 Text). The modified payoff matrix between *IN*_2_ and *IN*_1_ is

$$\begin{array}{c} \begin{array}{cc} & \begin{array}{cc} IN_{2} & IN_{1} \end{array}\\ \begin{array}{c} IN_{2}\\ IN_{1} \end{array} & \begin{array}{cc} a^{\prime } & b^{\prime }\\ c^{\prime } & d^{\prime } \end{array} \end{array}=\begin{array}{cc} & \begin{array}{cc} IN_{2} & IN_{1} \end{array}\\ \begin{array}{c} IN_{2}\\ IN_{1} \end{array} & \begin{array}{cc} 1 & \left(1-p_{2})(1+\Delta \right)\\ p_{2}(1+\Delta ) & 1 \end{array} \end{array}. \end{array}$$ (2)

From above modified payoff matrix, one can find that *b*′ − *c*′ = (1 − 2*p*_2_)(1 + Δ) is increased with respect to Δ (except when $p_{2}=\frac{1}{2}$), which means players with egalitarian norm *IN*_2_ gain greater advantage when interacting with players with less egalitarian norm *IN*_1_. Proposing low offers increases the chance of rejection, which leads to the reduction of the overall size of resources with the associated Ultimatum Game and thus decreases the payoffs of the proposers. Giving high offers is costly, but the cost is offset by obtaining a resource with an enlarged size. As the average offer level in the population increases, so does the optimal acceptance threshold value below which the offers proposed by relatively unfair individuals are rejected. This in turn favors even higher average offer level. Such a positive feedback mechanism holds the key to understanding the decisive role of resource management in the evolution of egalitarian social norm.

### Coevolution of egalitarian social norm and resource management

**Fig 4:**
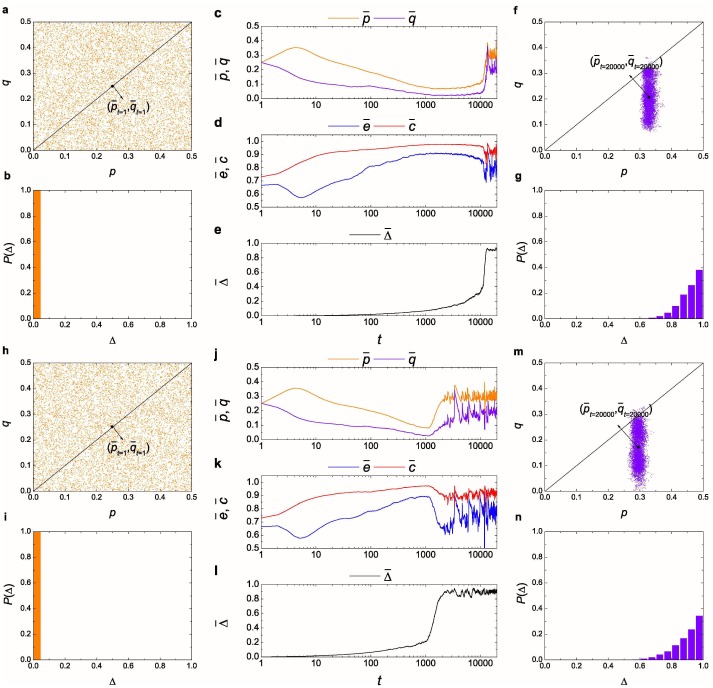
Coevolution of egalitarian social norm and resource management. Besides the evolution of norm, natural selection of resource management is implemented via the application of a pairwise comparison process to the individual resource management parameters Δ_*i*_. The time scale *τ*_*r*_ of resource management evolution is either much larger than the time scale *τ*_*n*_ of norm evolution (i.e., *s* = 0.1, top rows) or the same as *τ*_*n*_ (i.e., *s* = 1, bottom rows). The population is initialized with Δ_*i*_ = 0. During the evolutionary process, there is some perturbation off in the updating process, which follows a uniform distribution ranging from −0.025 to 0.025. For reasonability of the coevolutionary model, we assume that the intensity of resource management performed by two parties (e.g., individual *i* and *j*) is in accordance with Δ = min(Δ_*i*_, Δ_*j*_). Note that it is the most unfavorable situation for the evolution of resource management. (**a**, **h**) Typical norm distribution of the initial state (**a**: ${\bar{p}}_{t=1}\approx 0.25$, ${\bar{q}}_{t=1}\approx 0.25$, ${\bar{e}}_{t=1}\approx 0.667$, ${\bar{c}}_{t=1}\approx 0.73$; **h**: ${\bar{p}}_{t=1}\approx 0.249$, ${\bar{q}}_{t=1}\approx 0.252$, ${\bar{e}}_{t=1}\approx 0.664$, ${\bar{c}}_{t=1}\approx 0.73$). (**b**, **i**) Probability distribution of resource management in the initial state (**b**: ${\bar{\Delta }}_{t=1}=0$; **i**: ${\bar{\Delta }}_{t=1}=0$). (**c**, **d**, **e**, **j**, **k**, **l**) Time evolution of five population variables. (**f**, **m**) Typical norm distribution of the stationary state (**f**: ${\bar{p}}_{t=20,000}\approx 0.329$, ${\bar{q}}_{t=20,000}\approx 0.207$, ${\bar{e}}_{t=20,000}\approx 0.756$, ${\bar{c}}_{t=20,000}\approx 0.925$; **m**: ${\bar{p}}_{t=20,000}\approx 0.295$, ${\bar{q}}_{t=20,000}\approx 0.172$, ${\bar{e}}_{t=20,000}\approx 0.755$, ${\bar{c}}_{t=20,000}\approx 0.915$). (**g**, **n**) Probability distribution of resource management in the stationary state (**g**: ${\bar{\Delta }}_{t=20,000}\approx 0.914$; **n**: ${\bar{\Delta }}_{t=20,000}\approx 0.898$). Note the logarithmic scale on *x*-axis in (**c**, **d**, **e**, **j**, **k**, **l**). All other model setups are the same as those used in Fig 1.

Our results, therefore, suggest that resource management plays a determinant role in the evolution of egalitarian social norm. Then one may ask whether resource management itself is evolutionarily favored by natural selection in the long run. To explore this idea, we introduce the following extension to our model. Instead of assigning a fixed intensity of resource management, we start by a population consisting of individuals without the capability of resource management (i.e., Δ_*i*_ = 0). Then according to an endogenous evolutionary process, the resource management parameters Δ_*i*_ are subjected to evolution as well. To be specific, natural selection of the resource management parameters Δ_*i*_ is executed through using a pairwise comparison process [43, 44]. After every individual updates its norm, one randomly chosen individual copies another randomly selected neighbor’s resource management level with a probability proportionally to their payoff difference. This updating process is repeated *s* × *N* times before another round of games and norm updates takes place. Here *s* = *τ*_*n*_/*τ*_*r*_ represents the time scale ratio of norm evolution (i.e., *τ*_*n*_) to resource management evolution (i.e., *τ*_*r*_) [45]. An important issue then naturally arises: how fast resource management evolution happens relatively to norm evolution [46, 47]? If *τ*_*r*_ ≫ *τ*_*n*_, resource management evolution is more likely to be governed by genetic inheritance, whereas *τ*_*r*_ ≈ *τ*_*n*_ points to cultural imitation. Fig 4 displays the coevolutionary process of resource management and egalitarian social norm, when resource management evolution is modeled as either genetic inheritance (i.e., *s* = 0.1; see the top rows of Fig 4) or cultural imitation (i.e., *s* = 1; see the bottom rows of Fig 4). Notably, the stationary probability distribution of resource management converges to similar values for both cases, falling into the range that creates the most favorable environment for the evolution of egalitarian social norm (${\bar{p}}_{t=20,000}\approx 0.329$, ${\bar{q}}_{t=20,000}\approx 0.207$, ${\bar{e}}_{t=20,000}\approx 0.756$, ${\bar{c}}_{t=20,000}\approx 0.925$, ${\bar{\Delta }}_{t=20,000}\approx 0.914$ for *s* = 0.1; ${\bar{p}}_{t=20,000}\approx 0.295$, ${\bar{q}}_{t=20,000}\approx 0.172$, ${\bar{e}}_{t=20,000}\approx 0.755$, ${\bar{c}}_{t=20,000}\approx 0.915$, ${\bar{\Delta }}_{t=20,000}\approx 0.898$ for *s* = 1; see Fig 4). Such a finding has a very clear and intriguing implication: resource management that facilities a population with widespread fairness, empathy and collective conformity can be evolutionarily selected.

## Discussion

In this article, we have investigated the evolution of egalitarian social norm by resource management. The motivation of resource management is exclusively based on the canonical assumption that individuals are rational and selfish. Surprisingly, it was found that without any priori assumption about other-regarding preference [7], the self-interested process of evolution can lead to the evolution of egalitarian social norm even in the framework of conventional (deterministic) evolutionary game theory, whenever resource management is introduced. Furthermore, it is worth noting that the evolutionary mechanism of resource management does not fall into the category of direct reciprocity [48]. Direct reciprocity, which follows the principle: ‘I scratch your back and you scratch mine’, is a mechanism for the evolution of cooperation based on the repeated encounters between the same two individuals [49]. The standard framework of direct reciprocity is the iterated game (i.e., the repeated Prisoner’s Dilemma), while in our study individuals participate in the one-round game (i.e., the one-shot Ultimatum Game). In such a case, any two individuals can not reciprocate with each other in the form of direct reciprocity.

Previous theoretical studies aiming to model the evolution of social norms mainly fall into two categories [50]: (1) game-theoretical models and (2) opinion dynamics models. The game-theoretical models concentrate on the question of how the commitment to one specific norm can be reached in the presence of social dilemma, such as cooperation norm [39, 49, 51–54] or coordination norm [2, 55]. The opinion dynamics models attempt to understand how one of several possible behavior can establish a norm [56–58]. Different from these models, our present model naturally integrates the game-theoretical perspective with the opinion dynamics one. From the game-theoretical perspective, social norm is needed to keep the whole society in order [4], but unlikely, as it requires individuals to overcome selfish behavior. In our model, the evolution of egalitarian social norm constitutes such a dilemma. For any given self-compatible social norm $\left[\bar{p},\bar{q}\right]$, it is always beneficial for self-interested individuals to violate the norm by decreasing both the offer level *p* (as long as $p\geq \bar{q}$) and the acceptance threshold *q*. However, violation of the social norm may pose conflicts of interest between individuals, which leads to social disorder and instability. On the other hand, our model also generates the consensus process of individual norms from initial diversity to ultimate uniformity, a viewpoint of opinion dynamics. Therefore, the evolution of egalitarian social norm, when resource management is integrated, is remarkable, inasmuch as its collective formation from diverse individual norms, its spontaneous emergence in a selfish and unfair world and also its self-organized maintenance despite the challenge from norm violators.

In addition, our results might provide an explanation for the comprehension of egalitarian behavior existing across from human society to nonhuman animal world. Egalitarian motives in human adults are ubiquitous [6] and are also found consistent evidence in children and even infants very recently [59–61]. Nonhuman animals often have less strong exhibition in aversion to inequitable outcomes [62–64]. Some empirical studies have found strong reactions by animals towards inequity, though such affection is also dependent on the social status [65], social closeness [66], etc. On the other hand, some other experimental works found that chimpanzees are self-interested rational maximizers and are insensitive to fairness as their lack of refusals to unfair offers [67–69]. Given the facts that nonhuman animals and humans both exhibit fair behavior, but humans perform it more pervasive and are more efficient at managing resources, we may draw the conclusion that humans and nonhuman animals are at different stages in the efficiency of resource management. Such a conclusion can be further supported by considering the ethnographic observations that human beings are more egalitarian when they are more integrated into market activities [20, 70, 71]. This in turn suggests that the capability of resource management has coevolved with our egalitarian motives and could have a long evolutionary history with the resource management of nonhuman animals representing stages in the evolutionary process of the advanced resource management exhibited by humans. Although available ethnographic evidence is strongly pointing towards our findings, further empirical experiments are still required to test the predicted positive correlation between resource management and egalitarian social norm in a laboratory or field setting.

Finally, as a pioneering study of the Ultimatum Game with resource management, the present work can be extended in numerous ways. One possibility would be to consider that individuals have longer memory, instead of merely one-step memory, of past outcomes of deals. Moreover, resource management in our model requires the following two procedures: information-storage procedure (i.e., memorizing the previous outcomes of deals) and information-processing procedure (i.e., adjusting resource allocation dependent on the memory). Naturally, another possibility would be to consider that this dual-step process of resource management is subject to errors. Work along these lines is in progress.

## Methods

### Model definition

Herein, we present details that give the definition of the model. The population structure is described by a network, where each node is occupied by an individual, and each edge denotes who interacts and competes with whom [44, 72–77]. In our model, each time step (e.g., at time step *t*) includes two consecutive phases: individual interaction phase and norm updating phase.

#### Individual interaction phase

In the individual interaction phase, every individual plays the Ultimatum Game with each of its neighbors, once in the proposer role and once in the responder role. The individual norm of each player can be characterized by a vector *IN* = [*p*, *q*]^*T*^. The value of *p* denotes the fraction of the resource offered by the player when acting as a proposer, while the value of *q* indicates the acceptance threshold, i.e., the minimum fraction that the player accepts when acting as a responder. Based on rational self-interest, the two components *p* and *q* of each individual’s norm vector [*p*, *q*]^*T*^ are constraint within the interval [0, 0.5]. Let *P*(*IN*_*i*_, *IN*_*j*_) be the payoff that player *i* with individual norm *IN*_*i*_ = [*p*_*i*_, *q*_*i*_]^*T*^ gets from player *j* with individual norm *IN*_*j*_ = [*p*_*j*_, *q*_*j*_]^*T*^. Thus *P*(*IN*_*i*_, *IN*_*j*_) is given by

$$\begin{array}{c} P(IN_{i},IN_{j})=(1-p_{i})H(p_{i}-q_{j})R_{i}+p_{j}H(p_{j}-q_{i})R_{j}, \end{array}$$ (3)

where *R*_*i*_ (*R*_*j*_) is the amount of resource allocated to the Ultimatum Game, where player *i* (*j*) acts as a proposer, and *j* (*i*) as a responder. The Heaviside step function *H*(*x*) satisfies

$$\begin{array}{c} H(x)=\{\begin{array}{c} 0,\;\;\;\;\text{if}\;\;x<0\\ 1,\;\;\;\;\text{if}\;\;x\geq 0 \end{array}. \end{array}$$ (4)

**Fig 5:**
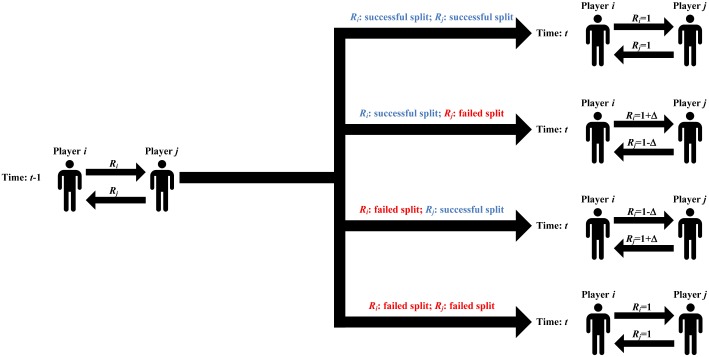
Schematic illustration of resource management. The player at the end of the small arrow plays as a proposer, while the one at the front of the small arrow acts as a responder for each Ultimatum Game. The size of resource allocated to each deal is denoted at the top of the small arrow. *R*_*i*_ (*R*_*j*_) is the resource to be split in one of the two Ultimatum Games played between player *i* and *j*, in which *i* (*j*) acts as the proposer, and *j* (*i*) as the responder. In our model of resource management, the allocation of resources in present deals (e.g., at time step *t*) is dependent on the outcome of previous ones (e.g., at time step *t* − 1).

In our model of resource management, resources are adaptively allocated to the deals. That is, the allocation of resources in present deals (e.g., at time step *t*) relies on the outcome of previous ones (e.g., at time step *t* − 1). Herein, we assume that both parties have the autonomy of making the decision on how to allocate resources to the two deals negotiated between them. To avoid a waste of resources, both parties incline to allocate more resources to the deal, where an agreement was reached, and less resources to the deal, where an agreement was broken. Based on the four possible outcomes of the two deals negotiated by two individuals, e.g., *i* and *j*, at time step *t* − 1, we assume that both individuals adopt the following resource management scheme at time step *t* (see Fig 5):

1. If both *R*_*i*_ and *R*_*j*_ are successfully split between *i* and *j* at time step *t* − 1, *R*_*i*_ and *R*_*j*_ at time step *t* are given by *R*_*i*_ = 1 and *R*_*j*_ = 1, respectively.
2. If *R*_*i*_ is successfully split but *R*_*j*_ is not at time step *t* − 1, *R*_*i*_ and *R*_*j*_ at time step *t* are given by *R*_*i*_ = 1 + Δ and *R*_*j*_ = 1 − Δ, respectively.
3. If *R*_*j*_ is successfully split but *R*_*i*_ is not at time step *t* − 1, *R*_*i*_ and *R*_*j*_ at time step *t* are given by *R*_*i*_ = 1 − Δ and *R*_*j*_ = 1 + Δ, respectively.
4. If both *R*_*i*_ and *R*_*j*_ are failed to be split between *i* and *j* at time step *t* − 1, *R*_*i*_ and *R*_*j*_ at time step *t* are given by *R*_*i*_ = 1 and *R*_*j*_ = 1, respectively.

Here the parameter Δ measures the intensity of resource management (i.e., the extent to which players adaptively respond to the outcomes of the previous deals). For reasonability of our model, we set 0 ≤ Δ ≤ 1, ensuring that *R*_*i*_ ≥ 0 and *R*_*j*_ ≥ 0. Note that the rationality of equal allocation of resources lies in both players’ unawareness of which deal is better than the other one, when both *R*_*i*_ and *R*_*j*_ are succeed or failed to be split. It is also worth mentioning that the total size of resources *R*_*i*_ + *R*_*j*_ = 2 is constant, so that the total maximal payoff of the population is invariant with respect to Δ. After interacting with each player within its neighborhood, player *i* with individual norm *IN*_*i*_ = [*p*_*i*_, *q*_*i*_]^*T*^ obtains its average payoff, which is given by

$$\begin{array}{c} P_{IN_{i}}=\frac{\sum_{k\in \Gamma (i)}P(IN_{i},IN_{k})}{M_{i}}, \end{array}$$ (5)

where *M*_*i*_ is the number of players in player *i*’s neighborhood Γ(*i*).

#### Norm updating phase

Subsequent to the individual interaction phase, players synchronously update their norms, either by random exploration of available norms or by payoff-biased imitation of norms. With probability *μ* (the exploration rate), a player, e.g., *i*, switches to a randomly selected norm [78, 79]; with probability 1 − *μ*, the player experiences the norm imitation event based on a pairwise comparison process [43, 44]. Specifically, player *i* adopts the individual norm *IN*_*j*_ of a randomly selected neighbor *j* with the probability

$$\begin{array}{c} T(P_{IN_{j}}-P_{IN_{i}})=\frac{1}{1+\exp[-(P_{IN_{j}}-P_{IN_{i}})/K]}, \end{array}$$ (6)

where $P_{IN_{i}}$ and $P_{IN_{j}}$ are the average payoffs of *i* and *j*, respectively. The parameter *K* quantifies the amplitude of noise allowing the irrational choices [44]. As the individual norms are continuous, it is almost impossible to imitate the norm of the role model precisely. Thus we add a small perturbation to the process of norm imitation. Namely, after learning from *j*, the individual norm of *i* becomes *IN*_*i*_ = [*p*_*i*_ + *ε*_1_, *q*_*i*_ + *ε*_2_] with *ε*_1_ and *ε*_2_ being randomly picked up from the interval [−*ε*, *ε*]. Both the noise and the learning error are used to create a “trembling hand” effect [80]. After these updating events have been performed for all the individuals in the population, a new time step begins.

### Population variables

Here we introduce four population variables used for the description of collective behavior of a population.

1. Fairness: the mean offer level $\bar{p}$ and the mean acceptance level $\bar{q}$ of a population, which are respectively characterized by the average values of the offer level *p* and the acceptance threshold *q* of all players, that is,
  $$\begin{array}{c} \bar{p}=\sum_{i=1}^{N}p_{i}/N, \end{array}$$ (7)
  and
  $$\begin{array}{c} \bar{q}=\sum_{i=1}^{N}q_{i}/N, \end{array}$$ (8)
  where *N* is the population size, and *p*_*i*_ and *q*_*i*_ are the offer level and the acceptance threshold of player *i*, respectively. The mean offer level $0\leq \bar{p}\leq \frac{1}{2}$ and the mean acceptance level $0\leq \bar{q}\leq \frac{1}{2}$ of the population represent the fairness level of the whole population, and the larger values of $\bar{p}$ and $\bar{q}$ indicate the players are fairer in the population.
2. Empathy: the mean empathy level $\bar{e}$ of a population, which is given by
  $$\begin{array}{c} \bar{e}=\sum_{i=1}^{N}e_{i}/N, \end{array}$$ (9)
  where *N* is the population size, and *e*_*i*_ is the empathy level of *i*. Thus,
  $$\begin{array}{c} e_{i}=1-|p_{i}-q_{i}|/e, \end{array}$$ (10)
  where *e* = 0.5 is a normalization factor that bounds *e*_*i*_ in the range between zero and one, and *p*_*i*_ and *q*_*i*_ are the offer level and the acceptance threshold of player *i*, respectively. The parameter $\bar{e}$ characterizes the empathy level of the whole population, and the larger value of $\bar{e}$ indicates the players are more empathetic in the population.
3. Collective conformity: the conformity level of the individual norms that individuals of a population conform to, which is measured by
  $$\begin{array}{c} \bar{c}=\sum_{i=1}^{N}c_{i}/N, \end{array}$$ (11)
  where *N* is the population size, and *c*_*i*_ denotes the coherence between the individual norm of *i* and the social norm. Hence,
  $$\begin{array}{c} c_{i}=1-\sqrt{{\left(p_{i}-\bar{p}\right)}^{2}+{\left(q_{i}-\bar{q}\right)}^{2}}/c, \end{array}$$ (12)
  where $c=\sqrt{2}/2$ is a normalization factor that confines *c*_*i*_ into the range between zero and one, and *p*_*i*_ and *q*_*i*_ are the offer level and the acceptance threshold of player *i*, respectively. The parameter $\bar{c}$ characterizes the conformity level of the individual norms, and the larger value of $\bar{c}$ indicates that the social norm $\left(\bar{p},\bar{q}\right)$ is more widely accepted by the individuals in a population.

### Simulation details

The simulations are performed on a fully connected network with *N* = 10^4^ nodes. The two components *p* and *q* of each individual’s norm vector [*p*, *q*] are randomly initialized in the interval [0, 0.5] independently. At the beginning of evolution, the size of resource to be allocated between two parties of each Ultimatum Game is set to unity. Equilibrium $\bar{p}$, $\bar{q}$, $\bar{e}$ and $\bar{c}$ values are evaluated by averaging over 5 × 10^6^ time steps after a transient time of 5 × 10^6^ time steps. We confirm that runs for longer time periods do not affect the presented results.

## Supporting information

S1 Text The supporting information is organized as follows. Section 1 presents the numerical results showing that resource management can induce the emergence of egalitarian social norm in a selfish and unfair world. Section 2 provides evidences supporting that resource management can lead to the maintenance of egalitarian social norm despite the presence of norm violators. Section 3 reports that the main results are robust against numerous model assumptions. Section 4 gives a detailed mean-field analysis of a mini Ultimatum Game with resource management.
(PDF)

## References

[1] Hechter M, Opp KD. Social Norms. New York: Russell Sage Foundation; 2001.

[2] Bicchieri C. The Grammar of Society: The Nature and Dynamics of Social Norms. UK: Cambridge University Press; 2006.

[3] Elster J. The Cement of Society: A Study of Social Order. UK: Cambridge University Press; 1989.

[4] Hechter M, Horne C. Theories of Social Order: A Reader. Stanford: Stanford University Press; 2003.

[5] Fehr E, Fischbacher U. The nature of human altruism. Nature. 2003; 425: 785–791. 10.1038/nature02043 14574401

[6] Dawes CT, Fowler JH, Johnson T, McElreath R, Smirnov O. Egalitarian motives in humans. Nature. 2007; 446: 794–796. 10.1038/nature05651 17429399

[7] Fehr E, Schmidt KM. A theory of fairness, competition, and cooperation. Q J Econ. 1999; 114: 817–868. 10.1162/003355399556151

[8] Preston SD, de Waal FBM. Empathy: Its ultimate and proximate bases. Behav Brain Sci. 2002; 25: 1–71. 10.1017/s0140525x02000018 12625087

[9] Page KM, Nowak MA. Empathy leads to fairness. Bull Math Biol. 2002; 64: 1101–1116. 10.1006/bulm.2002.0321 12508533

[10] de Waal FBM. Putting the altruism back into altruism: The evolution of empathy. Annu Rev Psychol. 2008; 59: 279–300. 10.1146/annurev.psych.59.103006.093625 17550343

[11] Fehr E, Fischbacher U. Social norms and human cooperation. Trends Cogn Sci. 2004; 8: 185–190. 10.1016/j.tics.2004.02.007 15050515

[12] Sigmund K. Punish or perish? Retailation and collaboration among humans. Trends Ecol Evol. 2007; 22: 593–600. 10.1016/j.tree.2007.06.012 17963994

[13] Güth W, Schmittberger R, Schwarze B. An experimental analysis of ultimatum bargaining. J Econ Behav Org. 1982; 3: 367–388. 10.1016/0167-2681(82)90011-7

[14] Maynard Smith J. Evolution and the Theory of Games. UK: Cambridge University Press; 1982.

[15] Weibull JW. Evolutionary Game Theory. Cambridge, MA: MIT Press; 1995.

[16] Thaler RH. Anomalies: The ultimatum game. J Econ Perspect. 1988; 2: 195–206. 10.1257/jep.2.4.195

[17] Güth W, Tietz R. Ultimatum bargaining behavior: A survey and comparison of exprimental results. J Econ Psychol. 1990; 11: 417–449. 10.1016/0167-4870(90)90021-Z

[18] Roth AE, Prasnikar V, Okuno-Fujiwara M, Zamir S. Bargaining and market behavior in Jerusalem, Ljubljana, Pittsburg, and Tokyo: An experimental study. Am Econ Rev. 1991; 81: 1068–1095.

[19] Bolton GE, Zwick R. Anonymity versus punishment in ultimatum bargaining. Games Econ Behav. 1995; 10: 95–121. 10.1006/game.1995.1026

[20] Henrich J, Boyd R, Bowles S, Camerer C, Fehr E, Gintis H, et al In search of homo economicus: Behavioral experiments in 15 small-scale societies. Am Econ Rev. 2001; 91: 73–78. 10.1257/aer.91.2.73

[21] Camerer CF. Behavioral Game Theory: Experiments in Strategic Interaction. Princeton: Princeton University Press; 2003.

[22] Güth W, Kocher MG. More than thirty years of ultimatum bargaining experiments: Motives, variations, and a survey of the recent literature. J Econ Behav Org. 2014; 108: 396–409. 10.1016/j.jebo.2014.06.006

[23] Kirchsteiger G. The role of envy in ultimatum games. J Econ Behav Org. 1994; 25: 373–389. 10.1016/0167-2681(94)90106-6

[24] Bethwaite J, Tompkinson P. The ultimatum game and non-selfish utility functions. J Econ Psychol. 1996; 17: 259–271. 10.1016/0167-4870(96)00006-2

[25] Rubinstein A. Perfect equilibrium in a bargaining model. Econometrica. 1982; 50: 97–109. 10.2307/1912531

[26] Bolton GE, Ockenfels A. ERC: A theory of equity, reciprocity, and competition. Am Econ Rev. 2000; 90: 166–193. 10.1257/aer.90.1.166

[27] Gale J, Binmore KG, Samuelson L. Learning to be imperfect: The ultimatum game. Games Econ Behav. 1995; 8: 56–90. 10.1016/S0899-8256(05)80017-X

[28] Huck S, Oechssler J. The indirect evolutionary approach to explaining fair allocations. Games Econ Behav. 1999; 28: 13–24. 10.1006/game.1998.0691

[29] Nowak MA, Page KM, Sigmund K. Fairness versus reason in the ultimatum game. Science. 2000; 289: 1773–1775. 10.1126/science.289.5485.1773 10976075

[30] Rand DG, Tarnita CE, Ohtsuki H, Nowak MA. Evolution of fairness in the one-shot anonymous ultimatum game. Proc Natl Acad Sci USA. 2013; 110: 2581–2586. 10.1073/pnas.1214167110 23341593 PMC3574936

[31] Wang X, Chen X, Wang L. Random allocation of pies promotes the evolution of fairness in the ultimatum game. Sci Rep. 2014; 4: 4534. 24686840 10.1038/srep04534 PMC3971395

[32] Page KM, Nowak MA, Sigmund K. The spatial ultimatum game. Proc R Soc Lond B. 2000; 267: 2177–2182. 10.1098/rspb.2000.1266 PMC1690799 11413630

[33] Killingback T, Studer E. Spatial ultimatum games, collaborations and the evolution of fairness. Proc R Soc Lond B. 2001; 268: 1797–1801. 10.1098/rspb.2001.1697 PMC1088811 11522198

[34] Szolnoki A, Perc M, Szabó G. Defense mechanisms of empathetic players in the spatial ultimatum game. Phys Rev Lett. 2012; 109: 078701 10.1103/PhysRevLett.109.078701 23006406

[35] Szolnoki A, Perc M, Szabó G. Accuracy in strategy imitations promotes the evolution of fairness in the spatial ultimatum game. EPL. 2012; 100: 28005 10.1209/0295-5075/100/28005

[36] Wu T, Fu F, Zhang Y, Wang L. Adaptive role switching promotes fairness in networked ultimatum game. Sci Rep. 2013; 3: 1550. 23528986 10.1038/srep01550 PMC3607882

[37] Zhang Y, Fu F. Strategy intervention for the evolution of fairness. PLoS ONE. 2018; 13: e0196524 10.1371/journal.pone.0196524 29718977 PMC5931496

[38] Debove S, Baumard N, André JB. Models of the evolution of fairness in the ultimatum game: a review and classification. Evol Hum Behav. 2016; 37: 245–254. 10.1016/j.evolhumbehav.2016.01.001

[39] Axelrod R. The Evolution of Cooperation. New York: Basic Books; 1984.

[40] Wang Q, He N, Chen X. Replicator dynamics for public goods game with resource allocation in large populations. Appl Math Comput. 2018; 328: 162–170.

[41] United States International Trade Commission DataWeb [Website]; 2015. Available from: https://dataweb.usitc.gov.

[42] Hofbauer J, Sigmund K. Evolutionary Games and Population Dynamics. UK: Cambridge University Press; 1998.

[43] Blume LE. The statistical mechanics of strategic interactions. Games Econ Behav. 1993; 5: 387–424. 10.1006/game.1993.1023

[44] Szabó G, Fáth G. Evolutionary games on graphs. Phys Rep. 2007; 446: 97–216. 10.1016/j.physrep.2007.04.004

[45] Roca CP, Helbing D. Emergence of social cohesion in a model society of greedy, mobile individuals. Proc Natl Acad Sci USA. 2011; 108: 11370–11374. 10.1073/pnas.1101044108 21709245 PMC3136269

[46] Gintis H. The hitchhiker’s guide to altruism: Gene-culture coevolution and the internalization of norms. J Theor Biol. 2003; 220: 407–418. 10.1006/jtbi.2003.3104 12623279

[47] Richerson PJ, Boyd R. Not by Genes Alone: How Culture Transformed Human Evolution. Chicago: University of Chicago Press; 2008.

[48] Trivers RL. The evolution of reciprocal altruism. Q Rev Biol. 1971; 46: 35–57. 10.1086/406755

[49] Nowak MA. Five Rules for the Evolution of Cooperation. Science. 2006; 314: 1560–1563. 10.1126/science.1133755 17158317 PMC3279745

[50] Helbing D, Johansson A. Cooperation, norms, and revolutions: A unified game-theoretical approach. PLoS ONE. 2010; 5: e12530 10.1371/journal.pone.0012530 20967256 PMC2953489

[51] Jiang L, Li W, Wang Z. Multiple effect of social influence on cooperation in interdependent network. Sci Rep. 2015; 5: 14657 10.1038/srep14657 26423024 PMC4589778

[52] Chu C, Mu C, Liu J, Liu C, Boccaletti S, Shi L, Wang Z. Aspiration-based coevolution of node weights promotes cooperation in the spatial prisoner’s dilemma game. New J phys. 2019; 21: 063024 10.1088/1367-2630/ab0999

[53] Chu C, Hu X, Shen C, Li T, Boccaletti S, Shi L, Wang Z. Self-organized interdependence among populations promotes cooperation by means of coevolution. Chaos. 2019; 29: 013139 10.1063/1.5059360 30709109

[54] Liu C, Shi J, Li T, Liu J. Aspiration driven coevolution resolves social dilemmas in networks. Appl Math Comput. 2019; 342: 247–254.

[55] Skyrms B. The Stag-Hunt Game and the Evolution of Social Structure. UK: Cambridge University Press; 2004.

[56] Axelrod R. The dissemination of culture: A model with local convergence and global polarization. J Conflict Resolut. 1997; 41: 203–226. 10.1177/0022002797041002001

[57] Ehrlich PR, Levin SA. The evolution of norms. PLoS Biol. 2005; 3: e194 10.1371/journal.pbio.0030194 15941355 PMC1149491

[58] Couzin ID, Ioannou CC, Demirel G, Gross T, Torney CJ, Hartnett A, et al Uninformed individuals promote democratic consensus in animal groups. Science. 2011; 334: 1578–1580. 10.1126/science.1210280 22174256

[59] Schmidt MFH, Sommerville JA. Fairness expectations and altruistic sharing in 15-month-old human infants. PLoS ONE. 2011; 6: e23223 10.1371/journal.pone.0023223 22003380 PMC3188955

[60] Fehr E, Bernhard H, Rockenbach B. Egalitarianism in young children. Nature. 2008; 454: 1079–1083. 10.1038/nature07155 18756249

[61] Blake PR, McAuliffe K. ‘I had so much it didn’t seem fair’: Eight-year-olds reject two forms of inequity. Cognition. 2011; 120: 215–224. 10.1016/j.cognition.2011.04.006 21616483

[62] Brosnan SF, de Waal FBM. Monkeys reject unequal pay. Nature. 2003; 425: 297–299. 10.1038/nature01963 13679918

[63] Range F, Horn L, Viranyi Z, Huber L. The absence of reward induces inequity aversion in dogs. Proc Natl Acad Sci USA. 2009; 106: 340–345. 10.1073/pnas.0810957105 19064923 PMC2629244

[64] Proctor D, Williamson RA, de Waal FBM, Brosnan SF. Chimpanzees play the ultimatum game. Proc Natl Acad Sci USA. 2013; 110: 2070–2075. 10.1073/pnas.1220806110 23319633 PMC3568338

[65] Brosnan SF. Nonhuman species’ reactions to inequity and their implications for fairness. Soc Justice Res. 2006; 19: 153–185.

[66] Brosnan SF, Schiff HC, de Waal FBM. Tolerance for inequity may increase with social closeness in chimpanzees. Proc R Soc B. 2005; 272: 253–258. 10.1098/rspb.2004.2947 15705549 PMC1634968

[67] Jensen K, Hare B, Call J, Tomasello M. What’s in it for me? Self-regard precludes altruism and spite in chimpanzees. Proc R Soc B. 2006; 273: 1013–1021. 10.1098/rspb.2005.3417 16627288 PMC1560238

[68] Jensen K, Call J, Tomasello M. Chimpanzees are rational maximizers in an ultimatum game. Science. 2007; 318: 107–109. 10.1126/science.1145850 17916736

[69] Kaiser I, Jensen K, Call J, Tomasello M. Theft in an ultimatum game: Chimpanzees and bonobos are insensitive to unfairness. Biol Lett. 2012; 8: 942–945. 10.1098/rsbl.2012.0519 22896269 PMC3497113

[70] Henrich J, Boyd R, Bowles S, Camerer C, Fehr E, Gintis H. Foundations of Human Sociality: Economic Experiments and Ethnographic Evidence from Fifteen Small-scale Societies. Oxford: Oxford University Press; 2004.

[71] Henrich J, Ensminger J, McElreath R, Barr A, Barrett C, Bolyanatz A, et al Markets, religion, community size, and the evolution of fairness and punishment. Science. 2010; 327: 1480–1484. 10.1126/science.1182238 20299588

[72] Santos FC, Pacheco JM. Scale-free networks provide a unifying framework for the emergence of cooperation. Phys Rev Lett. 2005; 95: 098104 10.1103/PhysRevLett.95.098104 16197256

[73] Ohtsuki H, Hauert C, Lieberman E, Nowak MA. A simple rule for the evolution of cooperation on graphs and social networks. Nature. 2006; 441: 502–505. 10.1038/nature04605 16724065 PMC2430087

[74] Fu F, Wang L, Nowak MA, Hauert C. Evolutionary dynamics on graphs: Efficient method for weak selection. Phys Rev E. 2009; 79: 046707 10.1103/PhysRevE.79.046707 PMC2735202 19518380

[75] Nowak MA, Tarnita CE, Antal T. Evolutionary dynamics in structured populations. Phil Trans R Soc B. 2010; 365: 19–30. 10.1098/rstb.2009.0215 20008382 PMC2842709

[76] Perc M, Szolnoki A. Coevolutionary games–a mini review. BioSystems. 2010; 99: 109–125. 10.1016/j.biosystems.2009.10.003 19837129

[77] Wu B, Zhou D, Fu F, Luo Q, Wang L, Traulsen A. Evolution of cooperation on stochastic dynamical networks. PLoS ONE. 2010; 5: e11187 10.1371/journal.pone.0011187 20614025 PMC2894855

[78] Traulsen A, Hauert C, De Silva H, Nowak MA, Sigmund K. Exploration dynamics in evolutionary games. Proc Natl Acad Sci USA. 2009; 106: 709–712. 10.1073/pnas.0808450106 19124771 PMC2630064

[79] Sigmund K, De Silva H, Traulsen A, Hauert C. Social learning promotes institutions for governing the commons. Nature. 2010; 466: 861–863. 10.1038/nature09203 20631710

[80] Selten R. Reexamination of the perfectness concept for equilibrium points in extensive games. Int J Gam Theor. 1975; 4: 25–55. 10.1007/BF01766400

