# Supplementary material for: Evolution of egalitarian social norm by resource management
Source: PLoS One. 2020 Jan 30;15(1):e0227902. doi: 10.1371/journal.pone.0227902 (PMC6992006; doi:10.1371/journal.pone.0227902)
Supplement: S1 Text — Section 1 presents the numerical results showing that resource management can induce the emergence of egalitarian social norm in a selfish and unfair world. Section 2 provides evidences supporting that resource management can lead to the maintenance of egalitarian social norm despite the presence of norm violators. Section 3 reports that the main results are robust against numerous model assumptions. Section 4 gives a detailed mean-field analysis of a mini Ultimatum Game with resource management. (PDF) [file pone.0227902.s001.pdf]

Supporting Information for
"Evolution of Egalitarian Social Norm by Resource Management"

Xiaofeng Wang[1,2*], Xiaojie Chen[3] & Long Wang[4*]

August 25, 2019

1 Department of Automation, School of Information Science & Technology, Donghua University, Shanghai, China
2 Engineering Research Center of Digitized Textile & Apparel Technology, Ministry of Education, Donghua University, Shanghai, China
3 School of Mathematical Sciences, University of Electronic Science and Technology of China, Chengdu, China
4 Center for Systems and Control, Peking University, Beijing, China
* Correspondence and requests for materials should be addressed to X.F.W or L.W. (email: rokywang1987@gmail.com or longwang@pku.edu.cn).

Contents

1 Emergence of Egalitarian Social Norm in a Selfish and Unfair World 2

2 Maintenance of Egalitarian Social Norm Despite the Presence of Norm Violators 3

3 Results are Robust against Various Model Alternations 4
3.1 Network Topology 4
3.2 Updating Pattern 7
3.3 Evolutionary Dynamics 8
3.4 Definition of the Ultimatum Game 10
3.5 Norm Distribution Range 11

4 Evolutionary Dynamics of the Mini Ultimatum Game with Resource Management 12

1 Emergence of Egalitarian Social Norm in a Selfish and Unfair World

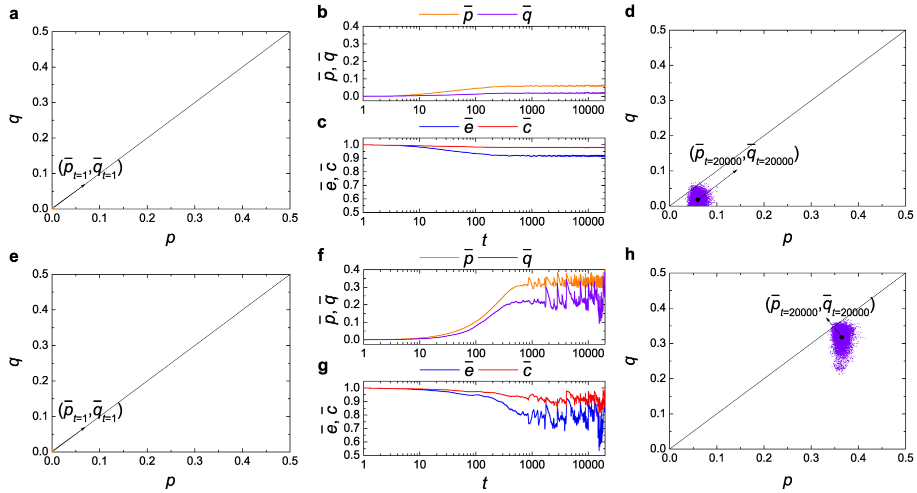

Figure S1: **Resource management induces the emergence of egalitarian social norm in a selfish and unfair world.** The top row is for $\Delta = 0$, and the bottom row is for $\Delta = 1$. (**a**, **e**) Characteristic norm distribution of the initial state (**a**: $\bar{p}_{t=1} = 0.001$, $\bar{q}_{t=1} = 0.001$, $\bar{e}_{t=1} = 1$, $\bar{c}_{t=1} = 1$; **e**: $\bar{p}_{t=1} = 0.001$, $\bar{q}_{t=1} = 0.001$, $\bar{e}_{t=1} = 1$, $\bar{c}_{t=1} = 1$). (**b**, **c**, **f**, **g**) Time evolution of four population variables. (**d**, **h**) Characteristic norm distribution of the equilibrium state (**d**: $\bar{p}_{t=20,000} \approx 0.06$, $\bar{q}_{t=20,000} \approx 0.018$, $\bar{e}_{t=20,000} \approx 0.917$, $\bar{c}_{t=20,000} \approx 0.979$; **h**: $\bar{p}_{t=20,000} \approx 0.364$, $\bar{q}_{t=20,000} \approx 0.318$, $\bar{e}_{t=20,000} \approx 0.907$, $\bar{c}_{t=20,000} \approx 0.97$). The simulations are performed on a fully connected network with $N = 10^4$ nodes. Initially, both p and q of each individual's norm vector $[p, q]$ are set to be 10^{-3}. Note the logarithmic scale on x-axis in (**b**, **c**, **f**, **g**). Parameter settings: exploration rate $\mu = 0$, noise level $K = 0.1$ and learning error range $\varepsilon = 5 \times 10^{-3}$.

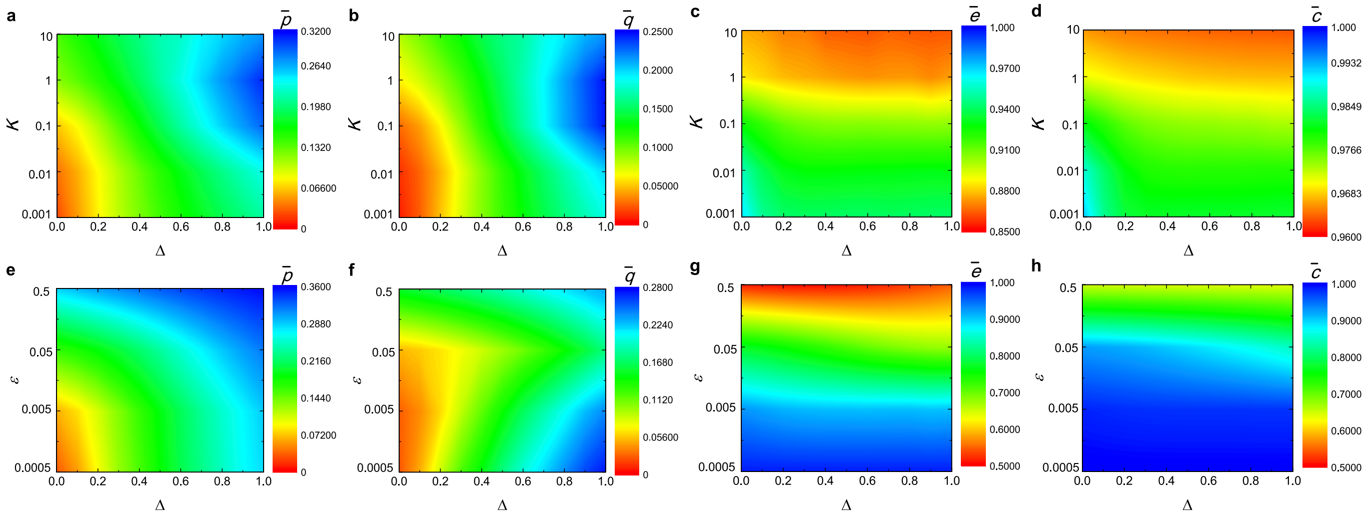

Figure S2: **Equilibrium state of a population in a selfish and unfair world.** The top (bottom) panels display the asymptotic values of four population variables describing the equilibrium state of the population as a function of Δ and the noise level K (the learning error range ε): (**a**, **b**, **e**, **f**) fairness (i.e., $\bar{p}$ and $\bar{q}$), (**c**, **g**) empathy (i.e., $\bar{e}$) and (**d**, **h**) collective conformity (i.e., $\bar{c}$). Equilibrium $\bar{p}$, $\bar{q}$, $\bar{e}$ and $\bar{c}$ values are evaluated by averaging over 5×10^6 time steps after a transient time of 5×10^6 time steps. All other model setups are the same as those used in Fig. S1.

In the main text, our results indicate that resource management plays a determinant role in the formation of egalitarian social norm. We now turn to evaluate the effect of resource management on the emergence of egalitarian social norm. Namely, whether the egalitarian social norm can arise in a selfish and unfair world in the first place. Fig. S1 shows the characteristic

collective behavior of a population on a fully connected network in a selfish and unfair world (i.e., with unfair individuals only in the population; $\bar{p}_{t=1} = 0.001$, $\bar{q}_{t=1} = 0.001$, $\bar{e}_{t=1} = 1$, $\bar{c}_{t=1} = 1$ for $\Delta = 0$; $\bar{p}_{t=1} = 0.001$, $\bar{q}_{t=1} = 0.001$, $\bar{e}_{t=1} = 1$, $\bar{c}_{t=1} = 1$ for $\Delta = 1$; see Fig. S1 **a** and **e**), when resource management is absent (i.e., $\Delta = 0$; see the top row of Fig. S1) and present (i.e., $\Delta = 1$; see the bottom row of Fig. S1). The evolutionary process shows that, without the help of resource management, the selfish and unfair social norm dominates the whole population ($\bar{p}_{t=20,000} \approx 0.06$, $\bar{q}_{t=20,000} \approx 0.018$, $\bar{e}_{t=20,000} \approx 0.917$, $\bar{c}_{t=20,000} \approx 0.979$; see Fig. S1 **b**, **c** and **d**). Conversely, with the aid of resource management, the selfish and unfair population is able to evolve into an egalitarian equilibrium state, where fairness emerges, and empathy as well as collective conformity maintain in a high level ($\bar{p}_{t=20,000} \approx 0.364$, $\bar{q}_{t=20,000} \approx 0.318$, $\bar{e}_{t=20,000} \approx 0.907$, $\bar{c}_{t=20,000} \approx 0.97$; see Fig. S1 **f**, **g** and **h**). It is worth mentioning that the initial appearance of fairness is possible only because of the presence of noise and learning error in the norm updating process. Afterwards, fairness is rapidly promoted by the population dynamics, as egalitarian individuals receive higher payoffs than unfair ones when combating with them (see Eq. (2) in the main text). We continue to find this significant effect of resource management, if the noise level or the learning error range is varied across the applicable span (see Fig. S2).

2 Maintenance of Egalitarian Social Norm Despite the Presence of Norm Violators

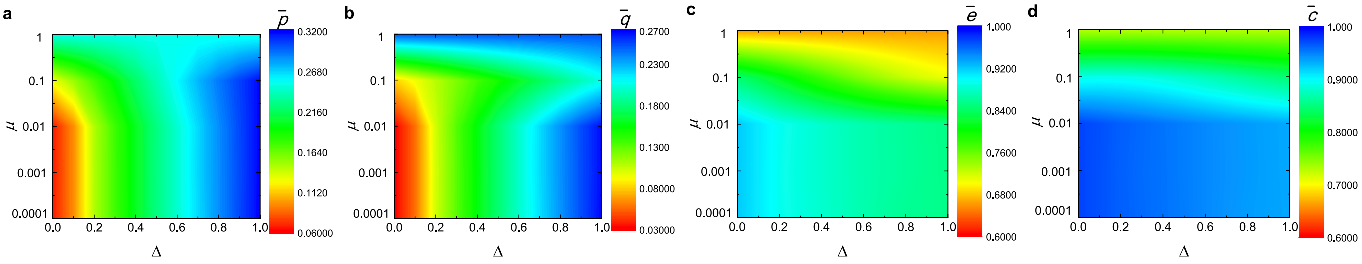

Figure S3: **Mutation-selection stationary state [1, 2] of a population in the presence of norm violators.** The panels display the stationary values of four population variables describing the mutation-selection stationary state of the population as a function of Δ and the exploration rate μ: (**a**, **b**) fairness (i.e., $\bar{p}$ and $\bar{q}$), (**c**) empathy (i.e., $\bar{e}$) and (**d**) collective conformity (i.e., $\bar{c}$). The simulations are performed on a fully connected network with $N = 10^4$ nodes. The stationary values of $\bar{p}$, $\bar{q}$, $\bar{e}$ and $\bar{c}$ are averaged over 10^7 time steps. The two components p and q of each individual's norm vector $[p, q]$ are randomly initialized in the interval $[0, 0.5]$ independently. Parameter settings: noise level $K = 0.1$ and learning error range $\varepsilon = 5 \times 10^{-3}$.

Both experimental and theoretical studies indicate that random exploration of available norms in cultural evolution, which is analogous to mutation in genetic evolution, is considerably common [1, 3]. Therefore, it is worth testing how resource management affects the maintenance of egalitarian social norm if it is challenged by norm violators (introduced by the exploration rate μ). To this end, we plot the stationary value of four population variables (i.e., $\bar{p}$, $\bar{q}$, $\bar{e}$ and $\bar{c}$) as a function of Δ and the exploration rate μ in Fig. S3. The population dynamics relies significantly on the exploration rate μ. In the low exploration rate limit $\mu \to 0$, a norm violator will either be eliminated or completely take over the whole population before another norm violator arises [4, 5, 6]. Thus, the population transitions between quasi-homogeneous states, in which all individuals in the population follow the similar norms at any time. In this case, norms that can resist against invasion do best (see Fig. S3). While in the high exploration rate limit $\mu \to 1$, all available norms are present at approximately equal abundances at the same time [1, 2]. Hence, the success of norms depends on their performance when playing against all norms with equal probability (see Fig. S3). Intermediate exploration rates lead to balanced outcomes between these two dynamical extremes. Comparison between the results obtained in the case of $\Delta = 0$ with those in $\Delta > 0$ reveals the sizable impact of resource management on the maintenance of egalitarian social norm. Despite the frequent challenge from norm violators, resource management helps the population adapt into a fairer state whereas empathy and collective conformity remain at a high level (see Fig. S3). This is because resource management can not only facilitate the egalitarian norms to protect themselves from invasion, but also improve their performance when competing with other norms simultaneously (see Eq. (2) in the main text).

3 Results are Robust against Various Model Alternations

Evolutionary game models may be quite sensitive to implementation details [7]. For this reason, we have examined numerous alternative model assumptions in this section. In all cases, the collective behavior of the evolutionary model is qualitatively the same (see the text below). Therefore, the main results remain unchanged within reasonable parameter variations and perturbations of the model.

3.1 Network Topology

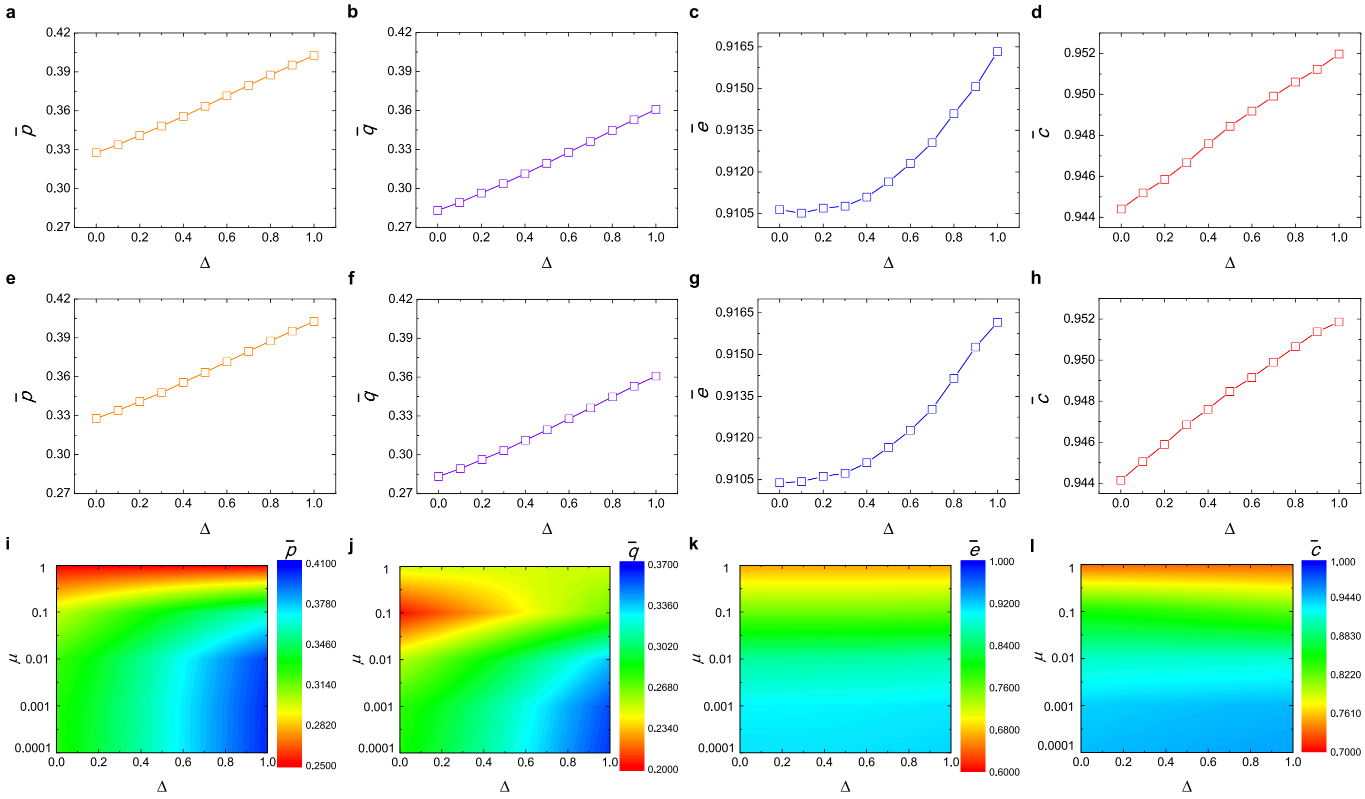

Figure S4: **Evolution of egalitarian social norm by resource management on square lattices with the *von Neumann* neighborhood (i.e., with average degree $\bar{z} = 4$).** (**a**, **b**, **c**, **d**) Formation of egalitarian social norm from diverse individual norms. (**e**, **f**, **g**, **h**) Emergence of egalitarian social norm in a selfish and unfair world. (**i**, **j**, **k**, **l**) Maintenance of egalitarian social norm despite the presence of norm violators. The top and middle rows display the asymptotic values of four population variables (i.e., fairness: $\bar{p}$ and $\bar{q}$, empathy: $\bar{e}$, and collective conformity: $\bar{c}$) describing the equilibrium state of the population as a function of Δ, whereas the bottom one shows the stationary values of four population variables as a function of Δ and the exploration rate μ. The applied square lattices are of the population size $N = 100 \times 100$ and with periodic boundary condition. Equilibrium $\bar{p}$, $\bar{q}$, $\bar{e}$ and $\bar{c}$ values for the top and middle rows are evaluated by averaging over 10^4 time steps after a transient time of 3×10^4 time steps, while stationary values of $\bar{p}$, $\bar{q}$, $\bar{e}$ and $\bar{c}$ for the bottom row are averaged over 4×10^4 time steps. To further increase the accuracy of our simulations, we have averaged the final outcome over 50 independent initial conditions. Parameter settings: exploration rate $\mu = 0$, noise level $K = 0.1$ and learning error range $\varepsilon = 5 \times 10^{-3}$ for the top and middle rows; noise level $K = 0.1$ and learning error range $\varepsilon = 5 \times 10^{-3}$ for the bottom row.

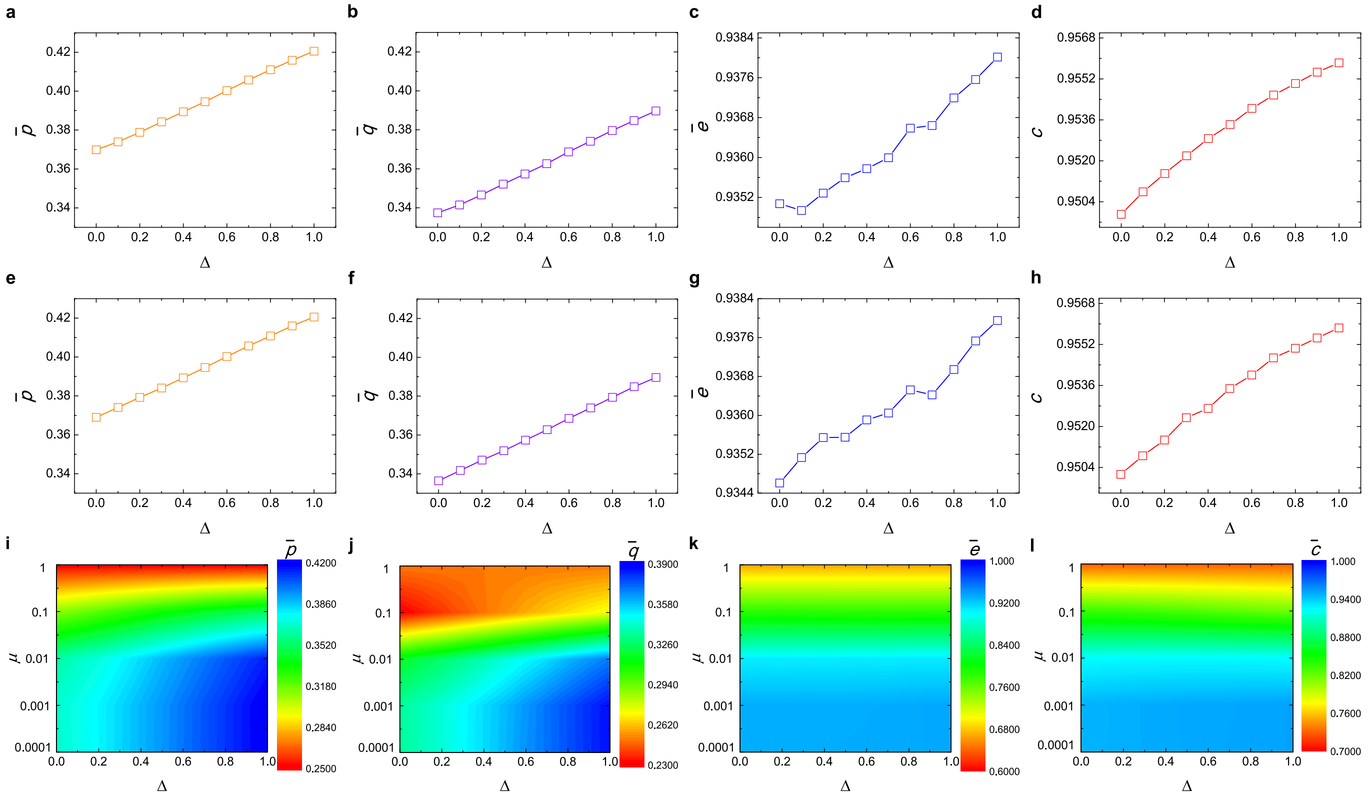

Figure S5: **Evolution of egalitarian social norm by resource management on small-world networks [8].** (**a**, **b**, **c**, **d**) Formation of egalitarian social norm from diverse individual norms. (**e**, **f**, **g**, **h**) Emergence of egalitarian social norm in a selfish and unfair world. (**i**, **j**, **k**, **l**) Maintenance of egalitarian social norm despite the presence of norm violators. The top and middle rows display the asymptotic values of four population variables (i.e., fairness: $\bar{p}$ and $\bar{q}$, empathy: $\bar{e}$, and collective conformity: $\bar{c}$) describing the equilibrium state of the population as a function of Δ, whereas the bottom one shows the stationary values of four population variables as a function of Δ and the exploration rate μ. The applied networks are of the population size $N = 1,000$, and the average connectivity is $\bar{z} = 4$. Equilibrium $\bar{p}$, $\bar{q}$, $\bar{e}$ and $\bar{c}$ values for the top and middle rows are evaluated by averaging over 2×10^4 time steps after a transient time of 2×10^4 time steps, while stationary values of $\bar{p}$, $\bar{q}$, $\bar{e}$ and $\bar{c}$ for the bottom row are averaged over 4×10^4 time steps. To further increase the accuracy of our simulations, the final outcome results from 10 different network realizations, with 50 independent initial strategy distributions for each realization. Parameter settings: exploration rate $\mu = 0$, noise level $K = 0.1$ and learning error range $\varepsilon = 5 \times 10^{-3}$ for the top and middle rows; noise level $K = 0.1$ and learning error range $\varepsilon = 5 \times 10^{-3}$ for the bottom row.

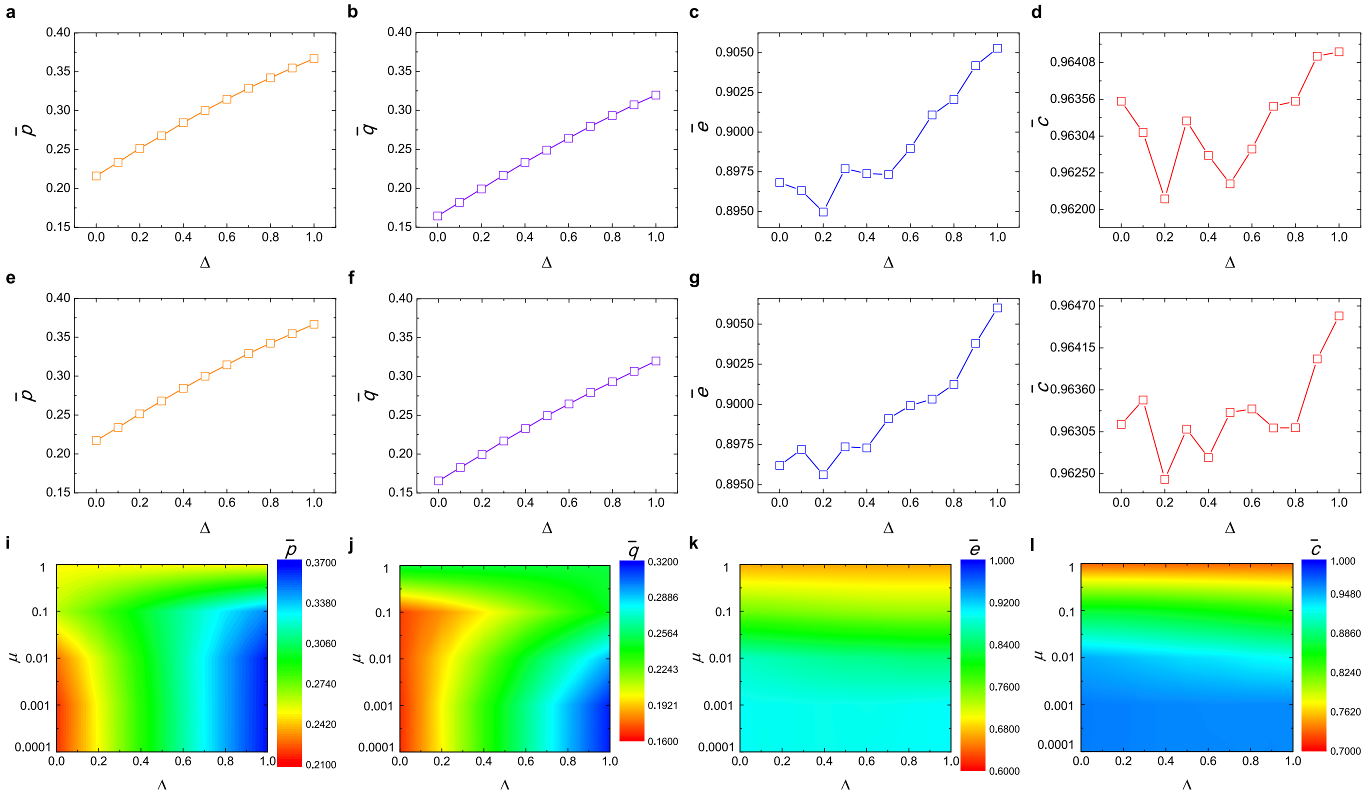

Figure S6: **Evolution of egalitarian social norm by resource management on scale-free networks [9].** (**a**, **b**, **c**, **d**) Formation of egalitarian social norm from diverse individual norms. (**e**, **f**, **g**, **h**) Emergence of egalitarian social norm in a selfish and unfair world. (**i**, **j**, **k**, **l**) Maintenance of egalitarian social norm despite the presence of norm violators. The top and middle rows display the asymptotic values of four population variables (i.e., fairness: $\bar{p}$ and $\bar{q}$, empathy: $\bar{e}$, and collective conformity: $\bar{c}$) describing the equilibrium state of the population as a function of Δ, whereas the bottom one shows the stationary values of four population variables as a function of Δ and the exploration rate μ. The applied networks are of the population size $N = 1,000$, and the average connectivity is $\bar{z} = 4$. Equilibrium $\bar{p}$, $\bar{q}$, $\bar{e}$ and $\bar{c}$ values for the top and middle rows are evaluated by averaging over 2×10^4 time steps after a transient time of 2×10^4 time steps, while stationary values of $\bar{p}$, $\bar{q}$, $\bar{e}$ and $\bar{c}$ for the bottom row are averaged over 4×10^4 time steps. To further increase the accuracy of our simulations, the final outcome results from 10 different network realizations, with 100 independent initial strategy distributions for each realization. Parameter settings: exploration rate $\mu = 0$, noise level $K = 0.1$ and learning error range $\varepsilon = 5 \times 10^{-3}$ for the top and middle rows; noise level $K = 0.1$ and learning error range $\varepsilon = 5 \times 10^{-3}$ for the bottom row.

In the main text, the equilibrium state of a population is evaluated for the populations on fully connected networks. Here we investigate how the results depend on network topology. To do this, we carried out computer simulations for square lattices, small-world networks [8] and scale-free networks [9], respectively (all with average degree $\bar{z} = 4$). Compared with the results in the main text, Figs. S4, S5 and S6 evidence that the behavior of the model is qualitatively the same. With the aid of "spatial reciprocity" [10], the spatial structured population evolves into a equilibrium state, which is more fair as well as empathetic, and individuals incline to conform to the egalitarian norm (see Fig. S4). The evolution of egalitarian social norm can be further facilitated, when individuals interact on small-world networks (compare Fig. S4 with Fig. S5). Namely, the small-world effect can further enhance the degree of social equity. Somewhat surprisingly, the heterogeneity of the degree distribution disfavors egalitarian social norm, as the levels of fairness, empathy and collective conformity achieved in the scale-free networks are more modest in comparison with those on square lattices (compare Fig. S4 with Fig. S6). This is in sharp contrast with other theoretical investigations that inhomogeneity of the networks can result in a remarkable boost in cooperation [11, 12].

3.2 Updating Pattern

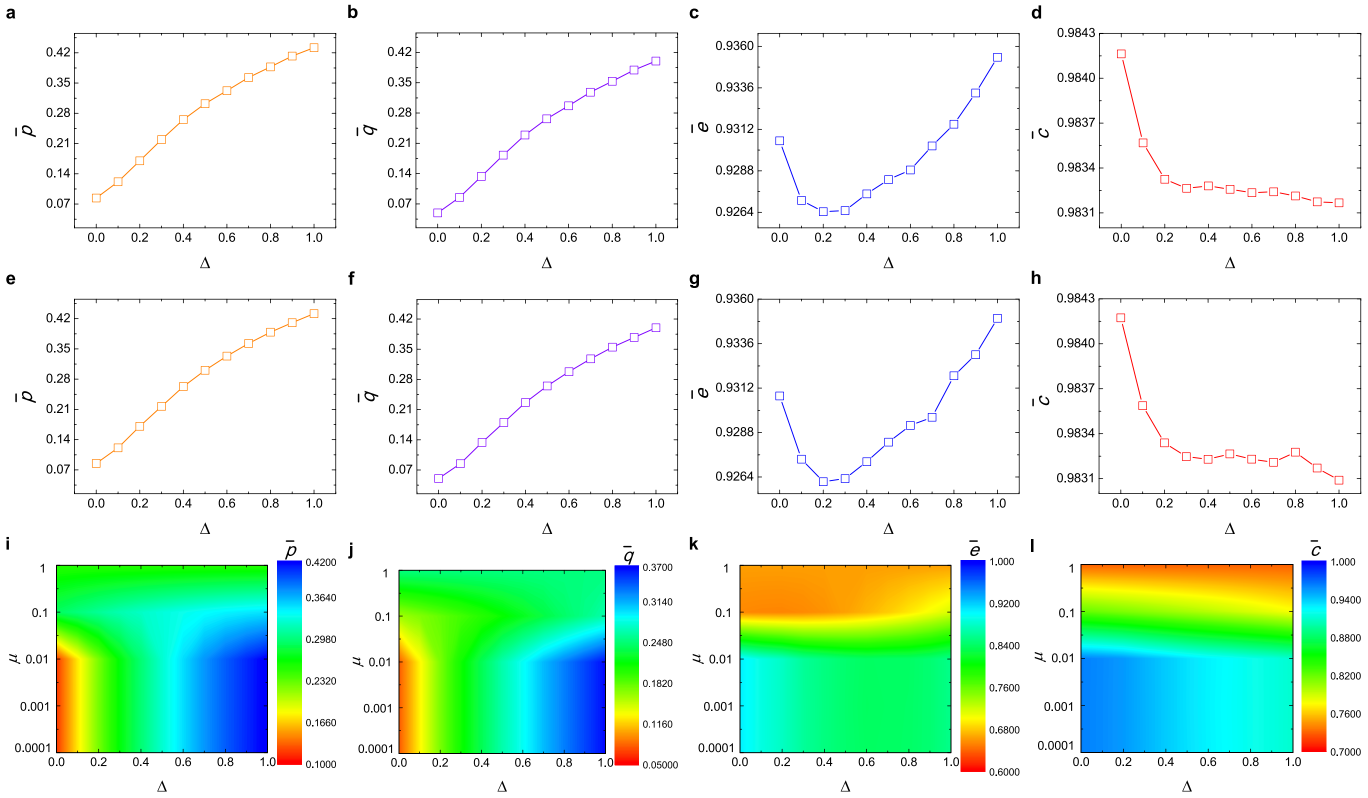

Figure S7: **Evolution of egalitarian social norm by resource management.** (**a**, **b**, **c**, **d**) Formation of egalitarian social norm from diverse individual norms. (**e**, **f**, **g**, **h**) Emergence of egalitarian social norm in a selfish and unfair world. (**i**, **j**, **k**, **l**) Maintenance of egalitarian social norm despite the presence of norm violators. The top and middle rows display the asymptotic values of four population variables (i.e., fairness: $\bar{p}$ and $\bar{q}$, empathy: $\bar{e}$, and collective conformity: $\bar{c}$) describing the equilibrium state of the population as a function of Δ, whereas the bottom one shows the stationary values of four population variables as a function of Δ and the exploration rate μ. Different from the synchronous updating rule in the main text, here the asynchronous updating rule is introduced into the population. In accordance with the asynchronous updating rule, each time step gives a chance once on average for every player to alter its strategy. The simulations are performed on a fully connected network with $N = 10^4$ nodes. Equilibrium $\bar{p}$, $\bar{q}$, $\bar{e}$ and $\bar{c}$ values for the top and middle rows are evaluated by averaging over 5×10^6 time steps after a transient time of 5×10^6 time steps, while stationary values of $\bar{p}$, $\bar{q}$, $\bar{e}$ and $\bar{c}$ for the bottom row are averaged over 10^7 time steps. Parameter settings: exploration rate $\mu = 0$, noise level $K = 0.1$ and learning error range $\varepsilon = 5 \times 10^{-3}$ for the top and middle rows; noise level $K = 0.1$ and learning error range $\varepsilon = 5 \times 10^{-3}$ for the bottom row.

Simulation reported in the main text employed synchronous updating pattern (i.e., non-overlapping generations). Instead, Fig. S7 presents results obtained via applying asynchronous updating (i.e., overlapping generations). In comparison with the results stated in the main text, we have found that such alternation does not change our results qualitatively. Applying overlapping generations (asynchronous updating) instead of nonoverlapping generations (synchronous updating) can further elevate the degree of social equity.

3.3 Evolutionary Dynamics

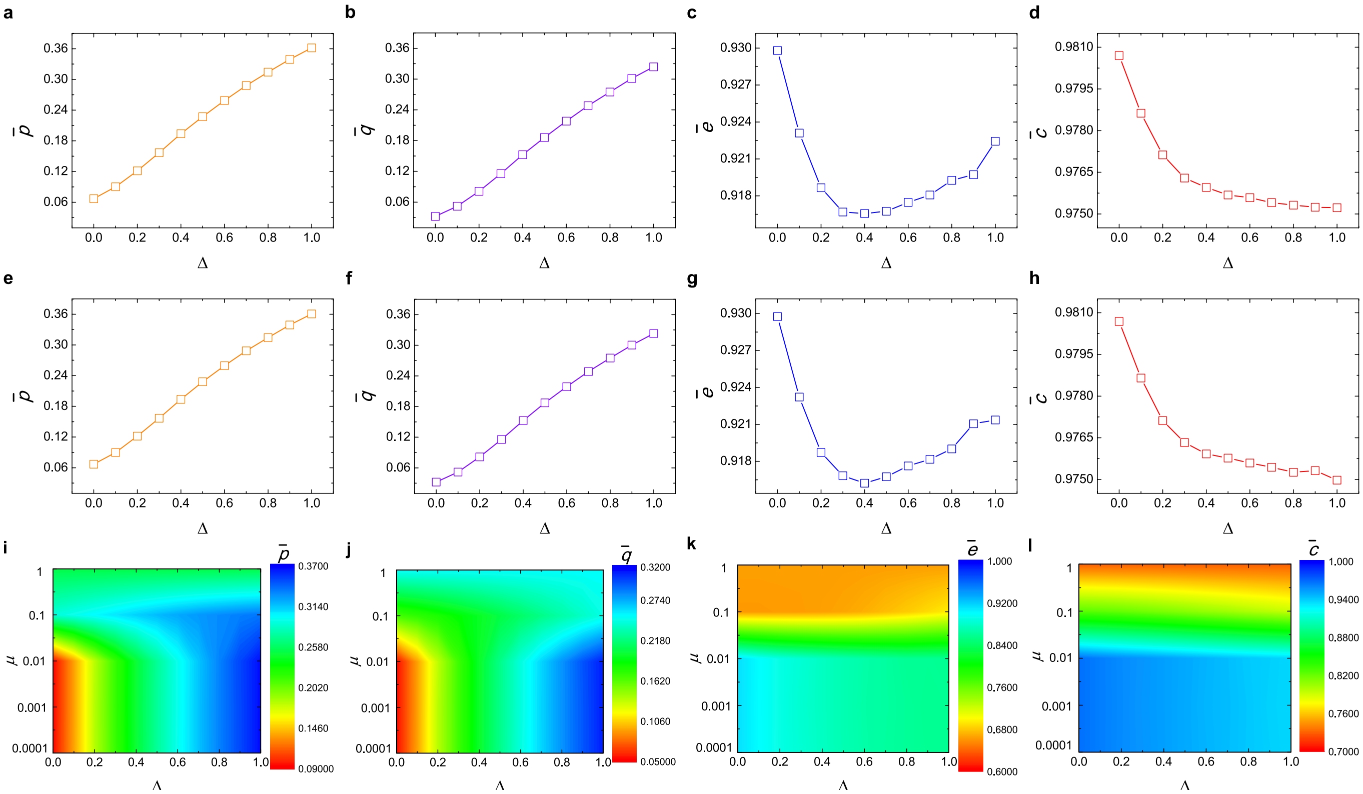

Figure S8: **Evolution of egalitarian social norm by resource management.** (**a**, **b**, **c**, **d**) Formation of egalitarian social norm from diverse individual norms. (**e**, **f**, **g**, **h**) Emergence of egalitarian social norm in a selfish and unfair world. (**i**, **j**, **k**, **l**) Maintenance of egalitarian social norm despite the presence of norm violators. The top and middle rows display the asymptotic values of four population variables (i.e., fairness: $\bar{p}$ and $\bar{q}$, empathy: $\bar{e}$, and collective conformity: $\bar{c}$) describing the equilibrium state of the population as a function of Δ, whereas the bottom one shows the stationary values of four population variables as a function of Δ and the exploration rate μ. Instead of the pairwise comparison process in the main text, here we adopt the birth-death process [13]. In each time step, one individual is selected for reproduction proportional to its fecundity $\exp[P]$, where P is the average payoff resulted from the interactions with other individuals; and the offspring replaces a randomly chosen individual. There is some perturbation off in the reproducing process, which follows a uniform distribution ranging from -0.005 to 0.005. The simulations are performed on a fully connected network with $N = 10^4$ nodes. Equilibrium $\bar{p}$, $\bar{q}$, $\bar{e}$ and $\bar{c}$ values for the top and middle rows are evaluated by averaging over 5×10^6 time steps after a transient time of 5×10^6 time steps, while stationary values of $\bar{p}$, $\bar{q}$, $\bar{e}$ and $\bar{c}$ for the bottom row are averaged over 10^7 time steps. Parameter settings: exploration rate $\mu = 0$, noise level $K = 0.1$ and learning error range $\varepsilon = 5 \times 10^{-3}$ for the top and middle rows; noise level $K = 0.1$ and learning error range $\varepsilon = 5 \times 10^{-3}$ for the bottom row.

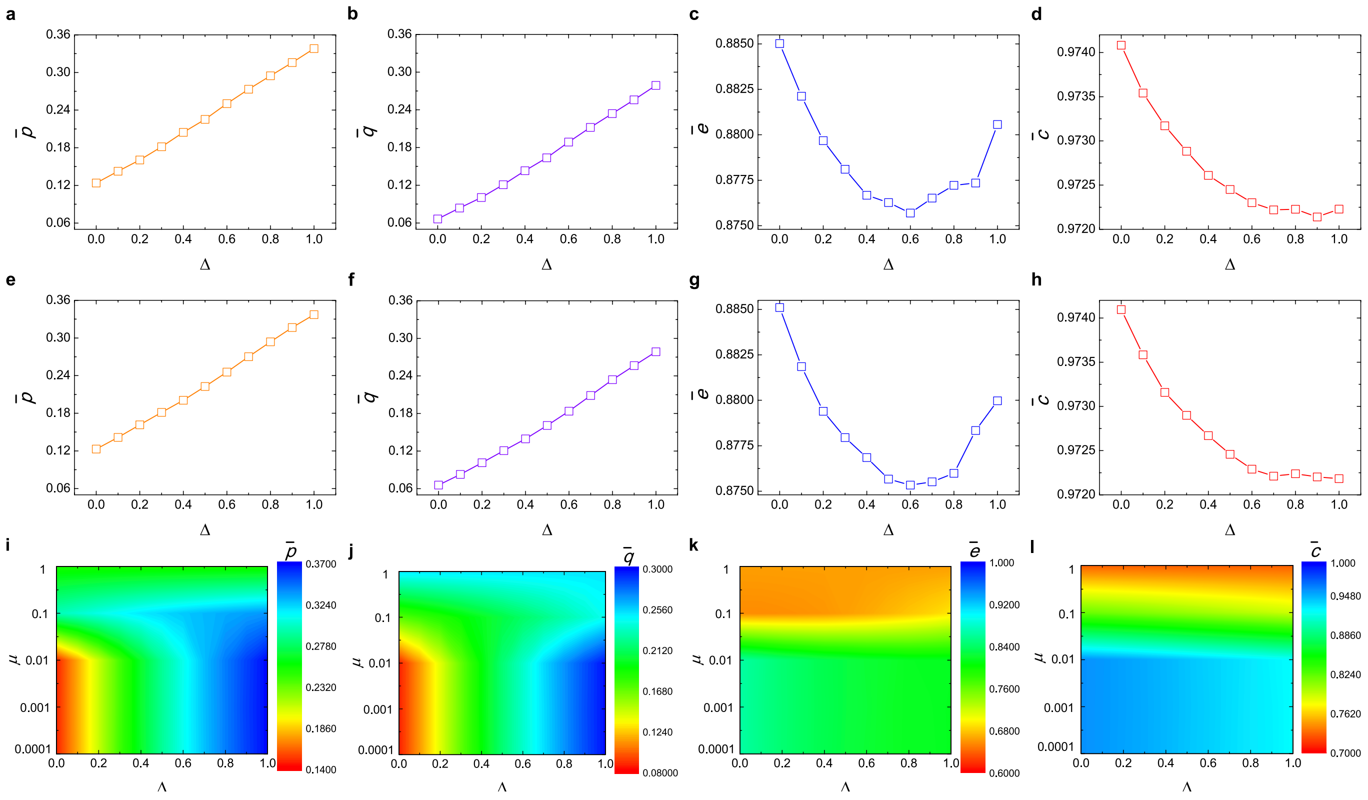

Figure S9: **Evolution of egalitarian social norm by resource management.** (**a**, **b**, **c**, **d**) Formation of egalitarian social norm from diverse individual norms. (**e**, **f**, **g**, **h**) Emergence of egalitarian social norm in a selfish and unfair world. (**i**, **j**, **k**, **l**) Maintenance of egalitarian social norm despite the presence of norm violators. The top and middle rows display the asymptotic values of four population variables (i.e., fairness: $\bar{p}$ and $\bar{q}$, empathy: $\bar{e}$, and collective conformity: $\bar{c}$) describing the equilibrium state of the population as a function of Δ, whereas the bottom one shows the stationary values of four population variables as a function of Δ and the exploration rate μ. Instead of the pairwise comparison process in the main text, here we adopt the death-birth process [14]. In each time step, a random individual is chosen to die; and its neighbors compete for the empty site proportional to their fecundity $\exp[P]$, where P is the average payoff resulted from the interactions with other individuals. There is some perturbation off in the reproducing process, which follows a uniform distribution ranging from -0.005 to 0.005. The simulations are performed on a fully connected network with $N = 10^4$ nodes. Equilibrium $\bar{p}$, $\bar{q}$, $\bar{e}$ and $\bar{c}$ values for the top and middle rows are evaluated by averaging over 5×10^6 time steps after a transient time of 5×10^6 time steps, while stationary values of $\bar{p}$, $\bar{q}$, $\bar{e}$ and $\bar{c}$ for the bottom row are averaged over 10^7 time steps. Parameter settings: exploration rate $\mu = 0$, noise level $K = 0.1$ and learning error range $\varepsilon = 5 \times 10^{-3}$ for the top and middle rows; noise level $K = 0.1$ and learning error range $\varepsilon = 5 \times 10^{-3}$ for the bottom row.

In our model, we have studied a pairwise comparison process in a finite population, which is naturally connected with the standard replicator dynamics in an infinite population [15]. One can envisage many different stochastic processes that describe evolutionary dynamics in finite populations, such as birth-death process [13] and death-birth process [14]. Results presented in Figs. S8 and S9 demonstrate that both stochastic processes produce similar collective behavior of the model as the pairwise comparison process does.

3.4 Definition of the Ultimatum Game

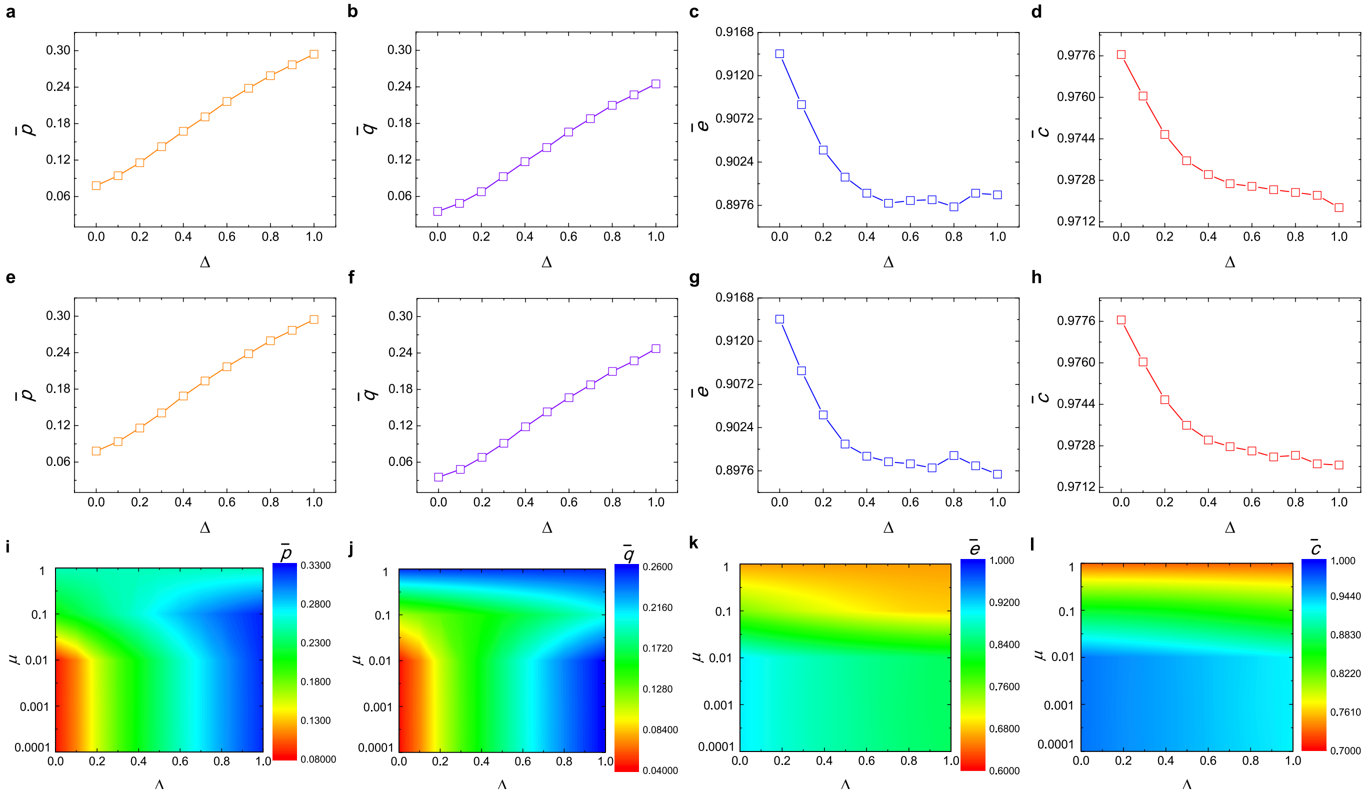

Figure S10: **Evolution of egalitarian social norm by resource management.** (**a**, **b**, **c**, **d**) Formation of egalitarian social norm from diverse individual norms. (**e**, **f**, **g**, **h**) Emergence of egalitarian social norm in a selfish and unfair world. (**i**, **j**, **k**, **l**) Maintenance of egalitarian social norm despite the presence of norm violators. The top and middle rows display the asymptotic values of four population variables (i.e., fairness: $\bar{p}$ and $\bar{q}$, empathy: $\bar{e}$, and collective conformity: $\bar{c}$) describing the equilibrium state of the population as a function of Δ, whereas the bottom one shows the stationary values of four population variables as a function of Δ and the exploration rate μ. Different from the definition of the Ultimatum Game in the main text, here the Ultimatum Game is played only once between the two parties, and roles (proposer or responder) are randomly assigned to them. The simulations are performed on a fully connected network with $N = 10^4$ nodes. Equilibrium $\bar{p}$, $\bar{q}$, $\bar{e}$ and $\bar{c}$ values for the top and middle rows are evaluated by averaging over 5×10^6 time steps after a transient time of 5×10^6 time steps, while stationary values of $\bar{p}$, $\bar{q}$, $\bar{e}$ and $\bar{c}$ for the bottom row are averaged over 10^7 time steps. Parameter settings: exploration rate $\mu = 0$, noise level $K = 0.1$ and learning error range $\varepsilon = 5 \times 10^{-3}$ for the top and middle rows; noise level $K = 0.1$ and learning error range $\varepsilon = 5 \times 10^{-3}$ for the bottom row.

In our definition of the Ultimatum Game, each individual plays the Ultimatum Game with each of its neighbors, once in the proposer role and once in the responder role. An alternative option is to randomly assign roles (proposer or responder) to the two parties and to assume that the Ultimatum Game is played only once between them. Fig. S10 shows that the qualitative outcome of the model is very similar to our case.

3.5 Norm Distribution Range

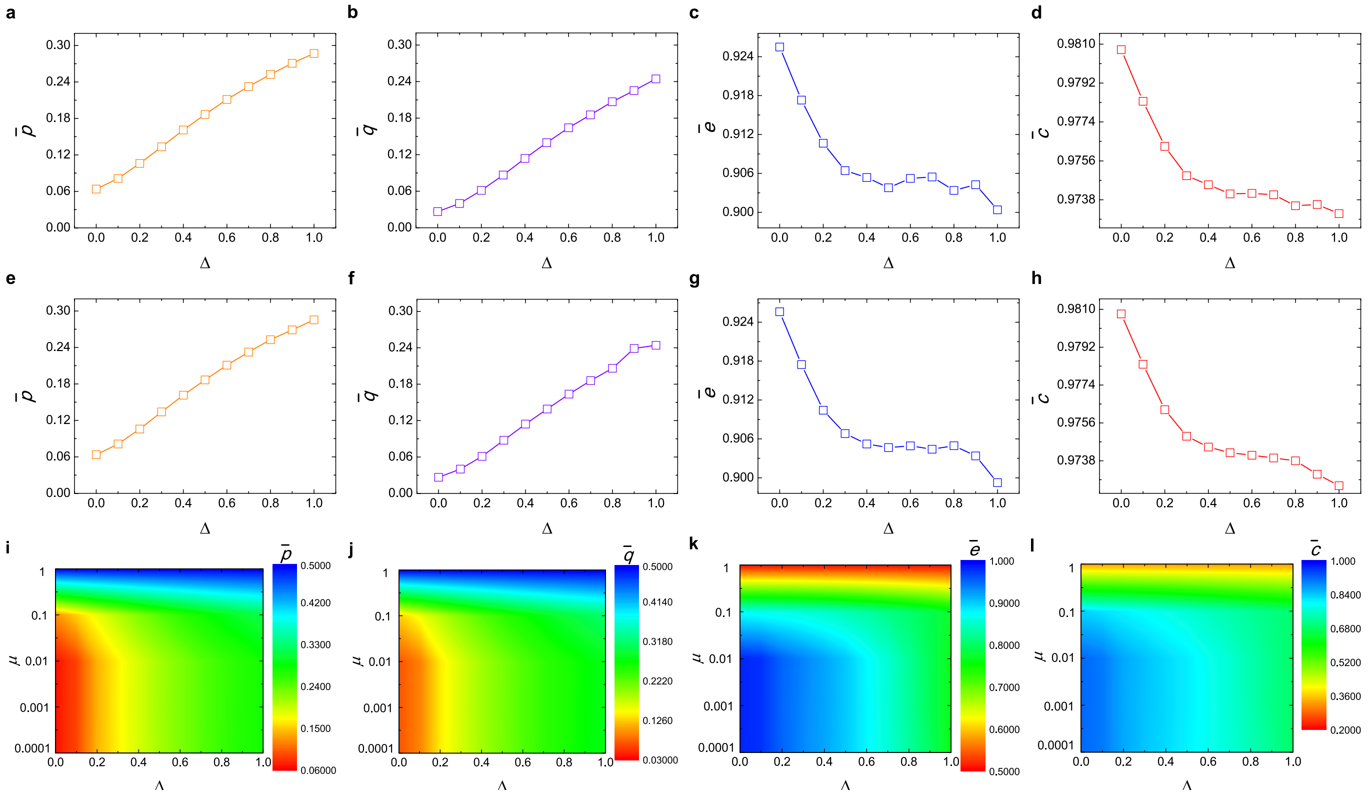

Figure S11: **Evolution of egalitarian social norm by resource management.** (**a**, **b**, **c**, **d**) Formation of egalitarian social norm from diverse individual norms. (**e**, **f**, **g**, **h**) Emergence of egalitarian social norm in a selfish and unfair world. (**i**, **j**, **k**, **l**) Maintenance of egalitarian social norm despite the presence of norm violators. The top and middle rows display the asymptotic values of four population variables (i.e., fairness: $\bar{p}$ and $\bar{q}$, empathy: $\bar{e}$, and collective conformity: $\bar{c}$) describing the equilibrium state of the population as a function of Δ, whereas the bottom one shows the stationary values of four population variables as a function of Δ and the exploration rate μ. Instead of the norm distribution range setup in the main text, here the domain of two components p and q of each individual's norm vector $[p,q]^T$ is the whole interval $[0,1]$. The simulations are performed on a fully connected network with $N = 10^4$ nodes. Equilibrium $\bar{p}$, $\bar{q}$, $\bar{e}$ and $\bar{c}$ values for the top and middle rows are evaluated by averaging over 5×10^6 time steps after a transient time of 5×10^6 time steps, while stationary values of $\bar{p}$, $\bar{q}$, $\bar{e}$ and $\bar{c}$ for the bottom row are averaged over 10^7 time steps. Parameter settings: exploration rate $\mu = 0$, noise level $K = 0.1$ and learning error range $\varepsilon = 5 \times 10^{-3}$ for the top and middle rows; noise level $K = 0.1$ and learning error range $\varepsilon = 5 \times 10^{-3}$ for the bottom row.

Based on rational self-interest, the two components p and q of each individual's norm vector $[p,q]^T$ are constraint within the interval $[0, 0.5]$ in our model. Another widely applied norm distribution range setup confines the two components p and q into the range $[0,1]$. Fig. S11 indicates that such change does not affect the generality of the reported results.

4 Evolutionary Dynamics of the Mini Ultimatum Game with Resource Management

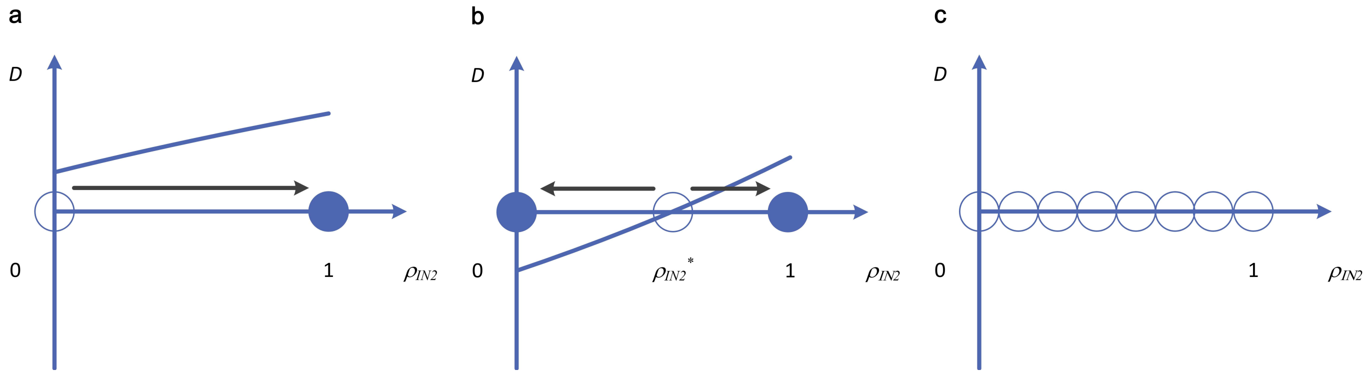

Figure S12: **Schematic presentation of the stable nature of the equilibria.** The solid dot represents the stable equilibrium, while the hollow one the unstable equilibrium. (**a**) IN_2 dominates IN_1. the equilibrium $\rho_{IN_2} = 1$ is stable, while the equilibrium $\rho_{IN_2} = 0$ are unstable. (**b**) IN_2 and IN_1 are bi-stable. Both the equilibrium $\rho_{IN_2} = 0$ and the equilibrium $\rho_{IN_2} = 1$ are stable, but the interior equilibrium $\rho_{IN_2} = \rho_{IN_2}{}^*$ is unstable. (**c**) IN_2 and IN_1 are neutral. Any state of the dynamical system is an unstable equilibrium.

In this section, we study how resource management affects the evolutionary dynamics of the mini Ultimatum Game, wherein only two individual norms $IN_1 = [p_1, q_1]$ and $IN_2 = [p_2, q_2]$ satisfying $0 \le q_1 \le p_1 < q_2 \le p_2 \le \frac{1}{2}$ are considered, on a fully connected network of infinite size. Here, we will show that the basin of attraction of norm IN_2 is enlarged with Δ. That is, the evolution of egalitarian norm can be facilitated by the introduction of resource management even in such unfairness-friendly conditions.

Assume there are a fraction $\rho_{IN_2}(t)$ ($\rho_{IN_2}(t-1)$) of players with norm IN_2 and $1 - \rho_{IN_2}(t)$ ($1 - \rho_{IN_2}(t-1)$) of players with norm IN_1 in an infinite population at time step t ($t-1$). Consequently, the average payoff of players with norm IN_2 at time step t is

$$P_{IN_2}(t) = \begin{cases} \rho_{IN_2}(t) + \frac{\rho_{IN_2}(t-1)\left[1-\rho_{IN_2}(t)\right](1-p_2)(1+\Delta)+\left[\rho_{IN_2}(t)-\rho_{IN_2}(t-1)\right]\left[1-\rho_{IN_2}(t)\right](1-p_2)}{\rho_{IN_2}(t)}, & \text{if } \rho_{IN_2}(t) - \rho_{IN_2}(t-1) \ge 0 \\ \rho_{IN_2}(t) + \left[\rho_{IN_2}(t-1) - \rho_{IN_2}(t)\right](1-p_2) + \left[1-\rho_{IN_2}(t-1)\right](1-p_2)(1+\Delta), & \text{if } \rho_{IN_2}(t) - \rho_{IN_2}(t-1) < 0 \end{cases}, \tag{S1}$$

whereas the average payoff of players with norm IN_1 at time step t is

$$P_{IN_1}(t) = \begin{cases} 1 - \rho_{IN_2}(t) + \left[\rho_{IN_2}(t) - \rho_{IN_2}(t-1)\right]p_2 + \rho_{IN_2}(t-1)\, p_2 (1+\Delta), & \text{if } \rho_{IN_2}(t) - \rho_{IN_2}(t-1) \ge 0 \\ 1 - \rho_{IN_2}(t) + \frac{\left[1-\rho_{IN_2}(t-1)\right]\rho_{IN_2}(t)p_2(1+\Delta)+\left[\rho_{IN_2}(t-1)-\rho_{IN_2}(t)\right]\rho_{IN_2}(t)p_2}{1-\rho_{IN_2}(t)}, & \text{if } \rho_{IN_2}(t) - \rho_{IN_2}(t-1) < 0 \end{cases}. \tag{S2}$$

Because the condition that $\rho_{IN_2}(t) = \rho_{IN_2}(t-1) = \rho_{IN_2}$ holds in the equilibrium, the average payoffs P_{IN_2} and P_{IN_1}, after the system reaches equilibrium, are respectively given by

$$P_{IN_2} = \rho_{IN_2} + (1 - \rho_{IN_2})(1 - p_2)(1 + \Delta), \tag{S3}$$

and

$$P_{IN_1} = 1 - \rho_{IN_2} + \rho_{IN_2} p_2 (1 + \Delta). \tag{S4}$$

The present norm updating rule (i.e., Eq. (6) in the main text) yields the following differential equation, which describes the motion for ρ_{IN_2} [16]:

$$\begin{aligned} &\frac{\partial \rho_{IN_2}}{\partial t} \\ &= \rho_{IN_2}(1-\rho_{IN_2})\left[T(P_{IN_2} - P_{IN_1}) - T(P_{IN_1} - P_{IN_2})\right] \\ &= -\rho_{IN_2}(1-\rho_{IN_2})\tanh\left[(P_{IN_1} - P_{IN_2})/2K\right]. \end{aligned} \tag{S5}$$

Obviously, there are two trivial equilibria, namely, $\rho_{IN_2} = 0$ and $\rho_{IN_2} = 1$. Let

$$D = P_{IN_2} - P_{IN_1} = \rho_{IN_2} (1 - \Delta) - p_2 + \Delta (1 - p_2). \tag{S6}$$

Then the derivative of D with respect to ρ_{IN_2} is given by

$$\frac{\partial D}{\partial \rho_{IN_2}} = 1 - \Delta. \tag{S7}$$

Because of $0 \leq \Delta \leq 1$, $\frac{\partial D}{\partial \rho_{IN_2}} \geq 0$ always holds, which indicates that D is non-decreased with respect to ρ_{IN_2}. Thus, depending on the sign of $D_{\rho_{IN_2}=0}$ and that of $D_{\rho_{IN_2}=1}$, we can classify three generic cases of evolutionary outcomes:

(i) IN_2 dominates IN_1 if $0 < p_2 \leq \frac{\Delta}{1+\Delta}$ (see Fig. S12 **a**).
As $0 < p_2 \leq \frac{\Delta}{1+\Delta}$, $D_{\rho_{IN_2}=0} \geq 0$ holds. Then the entire population will eventually evolve into a pure state consisting of players with norm IN_2. The only stable equilibrium is $\rho_{IN_2} = 1$. IN_2 is a strict Nash equilibrium and therefore is an evolutionarily stable strategy [17], while IN_1 is not (see Fig. S12 **a**). In this case, resource management can change the dynamical behavior of the system from bi-stability between IN_2 and IN_1 to complete dominance of IN_2 over IN_1. That is, the basin of attraction of norm IN_2 is enlarged to the whole domain.

(ii) IN_2 and IN_1 are bi-stable if $\frac{\Delta}{1+\Delta} < p_2 \leq \frac{1}{2}$ and $0 \leq \Delta < 1$ (see Fig. S12 **b**).
When $\frac{\Delta}{1+\Delta} < p_2 \leq \frac{1}{2}$ and $0 \leq \Delta < 1$, we have $D_{\rho_{IN_2}=0} < 0$ and $D_{\rho_{IN_2}=1} > 0$. Then both the equilibrium $\rho_{IN_2} = 0$ and the equilibrium $\rho_{IN_2} = 1$ are stable (see Fig. S12 **b**). IN_1 and IN_2 are both strict Nash equilibria and thus are evolutionarily stable strategies. In addition, there exists an interior fixed point (i.e., an interior equilibrium) for this dynamical system. Let $D = 0$, the interior fixed point ${\rho_{IN_2}}^*$ is given by

$${\rho_{IN_2}}^* = \frac{\Delta (p_2 - 1) + p_2}{1 - \Delta}. \tag{S8}$$

The stability of the interior fixed point can be determined by the sign of the derivative of D with respect to ρ_{IN_2}:

$$\frac{\partial D}{\partial \rho_{IN_2}} = 1 - \Delta. \tag{S9}$$

As $\Delta < 1$, $\frac{\partial D}{\partial \rho_{IN_2}} > 0$ always holds, which indicates that the interior fixed point ${\rho_{IN_2}}^*$ is unstable (see Fig. S12 **b**). Besides, we can also obtain the derivative of ${\rho_{IN_2}}^*$ with respect to Δ:

$$\frac{\partial {\rho_{IN_2}}^*}{\partial \Delta} = \frac{2p_2 - 1}{(1 - \Delta)^2}. \tag{S10}$$

As $p_2 \leq \frac{1}{2}$, $\frac{\partial {\rho_{IN_2}}^*}{\partial \Delta} \leq 0$ always holds, which indicates that ${\rho_{IN_2}}^*$ is non-increased with respect to Δ. Specifically, the basin of attraction of norm IN_2 is enlarged with Δ if $p_2 < \frac{1}{2}$, while the basin of attraction of IN_2 is invariant with Δ if $p_2 = \frac{1}{2}$.

(iii) IN_2 and IN_1 are neutral if $p_2 = \frac{1}{2}$ and $\Delta = 1$ (see Fig. S12 **c**).
If $p_2 = \frac{1}{2}$ and $\Delta = 1$, $D = 0$ always holds (see Eq. (S6)). Then natural selection will not change the composition of the population. Any mixture of IN_2 and IN_1 is an unstable equilibrium (see Fig. S12 **c**). In this case, the dynamic behavior of the system is unchanged with the variation of Δ.

From above analysis, we can find that for any given $p_2 < \frac{1}{2}$, resource management can help players with norm IN_2 to decrease the invasion barrier (see case (i) and (ii)) and thus promotes the evolution of egalitarian norm in such a mini Ultimatum Game.

Supplementary References

[1] Traulsen A, Hauert C, De Silva H, Nowak MA, Sigmund K. Exploration dynamics in evolutionary games. Proc Natl Acad Sci USA. 2009; 106: 709–712.

[2] Antal T, Traulsen A, Ohtsuki H, Tarnita CE, Nowak MA. Mutation-selection equilibrium in games with multiple strategies. J Theor Biol. 2009; 258: 614–622.

[3] Traulsen A, Semmann D, Sommerfeld RD, Krambeck HJ, Milinski M. Human strategy updating in evolutionary games. Proc Natl Acad Sci USA. 2010; 107: 2962–2966.

[4] Fudenberg D, Imhof LA. Imitation processes with small mutations. J Econ Theor. 2006; 131: 251–262.

[5] Hauert C, Traulsen A, Brandt H, Nowak MA, Sigmund K. Via freedom to coercion: The emergence of costly punishment. Science. 2007; 316: 1905–1907.

[6] Sigmund K, De Silva H, Traulsen A, Hauert C. Social learning promotes institutions for governing the commons. Nature. 2010; 466: 861–863.

[7] Roca CP, Cuesta JA, Sánchez A. Evolutionary game theory: Temporal and spatial effects beyond replicator dynamics. Phys Life Rev. 2009; 6: 208–249.

[8] Watts DJ, Strogatz SH. Collective dynamics of ‘small world’ networks. Nature. 1998; 393: 440–442.

[9] Barabási AL, Albert R. Emergence of scaling in random networks. Science. 1999; 286: 509–512.

[10] Page KM, Nowak MA, Sigmund K. The spatial ultimatum game. Proc R Soc Lond B. 2000; 267: 2177–2182.

[11] Santos FC, Pacheco JM. Scale-free networks provide a unifying framework for the emergence of cooperation. Phys Rev Lett. 2005; 95: 098104.

[12] Santos FC, Santos MD, Pacheco JM. Social diversity promotes the emergence of cooperation in public goods games. Nature. 2008; 454: 213–216.

[13] Nowak MA, Sasaki A, Taylor C, Fudenberg D. Emergence of cooperation and evolutionary stability in finite populations. Nature. 2004; 428: 646–650.

[14] Ohtsuki H, Hauert C, Lieberman E, Nowak MA. A simple rule for the evolution of cooperation on graphs and social networks. Nature. 2006; 441: 502–505.

[15] Traulsen A, Claussen JC, Hauert C. Coevolutionary dynamics: From finite to infinite populations. Phys Rev Lett. 2005; 95: 0238701.

[16] Szabó G, Vukov J, Szolnoki A. Phase diagrams for an evolutionary prisoner’s dilemma game on two-dimensional lattices. Phys Rev E. 2005; 72: 047107.

[17] Maynard Smith J, Price GR. The logic of animal conflict. Nature. 1973; 246: 15–18.
